# From Microplastics to Multifunctional Magnetic Nanomaterials: A Circular Strategy for Water Remediation

**DOI:** 10.3390/molecules31173117

**Published:** 2026-09-05

**Authors:** Rafael Herrera-Aquino, Sabino Veintemillas-Verdaguer, Fernando Agulló-Rueda, Fernanda Lyzeth Rivera, Nahuel Nuñez, Fernando Martín-Garrido, Helena Gavilán, Elin L. Winkler, María del Puerto Morales, Alvaro Gallo-Cordova

**Affiliations:** 1 Instituto de Ciencia de Materiales de Madrid, ICMM/CSIC, C/Sor Juana Inés de la Cruz 3, 28049 Madrid, Spain; sabino@icmm.csic.es (S.V.-V.); f.agullo.rueda@csic.es (F.A.-R.); 2 Facultad de Ingeniería Mexicali (FIM), Universidad Autónoma de Baja California, Av. Alvaro Obregón s/n, Mexicali 21100, BC, Mexico; fernanda.rivera.saldivar@uabc.edu.mx; 3 Departamento Magnetismo y Materiales Magnéticos, Gerencia de Física, Centro Atómico Bariloche, San Carlos de Bariloche 8400, Río Negro, Argentina; nahuel.nunez@ib.edu.ar (N.N.); elin.winkler@ib.edu.ar (E.L.W.); 4 Instituto de Nanociencia y Nanotecnología (CNEA CONICET), Nodo Bariloche, San Carlos de Bariloche 8400, Río Negro, Argentina; 5 Instituto Balseiro, CNEA-UNCuyo, Av. Bustillo, San Carlos de Bariloche 9500, Río Negro, Argentina; 6 Departamento de Química Física, Facultad de Ciencias Químicas, Universidad Complutense de Madrid (UCM), Ciudad Universitaria s/n, 28040 Madrid, Spain; femart10@ucm.es (F.M.-G.); hgavilan@ucm.es (H.G.)

**Keywords:** magnetic nanoparticles, iron oxide nanoparticles, microplastics, advanced oxidation processes, PET valorization, circular economy, environmental catalysis

## Abstract

Microplastic remediation strategies often overlook the management and valorization of the recovered waste, limiting their overall sustainability. Herein, we propose a closed-loop water remediation strategy in which polyethylene terephthalate microplastics (MicroPET) are not only removed from water but also converted into new magnetic nanomaterials for subsequent remediation cycles. MicroPET was initially harvested using magnetic iron oxide nanoflowers (NFs) and subsequently depolymerized by neutral hydrolysis, achieving an unscaled gravimetric PET mass conversion of 97%. Upon process scale-up and downstream purification, an isolated monomer yield of 28.7% was obtained for both purified terephthalic acid (TPA) and ethylene glycol (EG), as confirmed by ^1^H-NMR, FTIR, Raman, and osmometric analyses. The recovered supernatant from the unscaled hydrolysis was directly reused as the reaction medium for the microwave-assisted synthesis of maghemite magnetic iron oxide nanoparticles (MIONPs), producing bimodal single-core nanoparticles composed of 5 ± 1 and 29 ± 6 nm crystallites. Despite the absence of the multicore nanoflower architecture and the associated reduction in magnetic performance, the synthesized nanoparticles still demonstrated a remarkable MicroPET harvesting capacity of 1000 mg g^−1^ under optimized conditions (compared with 10,000 mg g^−1^ achieved by the original NFs). Furthermore, both the pristine nanoparticles and the MicroPET-loaded hybrid materials efficiently catalyzed methylene blue degradation through a heterogeneous Fenton-like process, with alternating magnetic field activation increasing the decolorization efficiency by ≈20% compared with room-temperature conditions. These results demonstrate that PET-derived EG can be directly reintegrated into the synthesis of functional magnetic nanomaterials, establishing a circular strategy that combines pollutant removal, plastic waste valorization, and catalytic water remediation.

## 1. Introduction

The global increase in anthropogenic activities has resulted in an unprecedented release of emerging contaminants into aquatic ecosystems [1,2,3], posing a threat to both biodiversity and human health. Among these contaminants, microplastics have attracted considerable attention due to their widespread occurrence, environmental persistence, and resistance to conventional wastewater treatment processes [4,5]. Poly(ethylene terephthalate) microplastics (MicroPET), defined as plastic particles with a diameter below 5 mm [6], are generated through the fragmentation and weathering of larger plastic debris. Over time, the MicroPET surface undergoes oxidation, generating hydroxyl and carboxyl groups [7], which alter MicroPET’s colloidal stability and facilitate particle aggregation [8], thereby affecting their transport and fate in water.

Current conventional water treatment technologies often fail to achieve complete removal, particularly for sub-millimetric particles and recalcitrant chemical compounds [9,10], necessitating the development of advanced materials that are not only efficient but also environmentally sustainable [11]. In this context, nanotechnology has emerged as a frontline solution. Specifically, MIONPs are particularly valued for their high surface-to-volume ratio, biocompatibility, and ease of magnetic separation [12]. Beyond their intrinsic physicochemical advantages, MIONPs can be readily functionalized with tailored surface chemistries, such as polymeric coatings, surfactants, or catalytic moieties, to enhance their affinity toward hydrophobic polymer fragments and associated contaminants [13]. This tunability enables effective adsorption and aggregation of MicroPET, facilitating their subsequent removal via external magnetic fields [14]. Moreover, recent studies have demonstrated that iron-based systems can be integrated with advanced oxidation processes (AOPs) or photocatalytic platforms, promoting not only the capture but also the partial degradation of microplastics into smaller, less persistent compounds [15,16]. Importantly, their magnetic recoverability allows repeated reuse, addressing key concerns regarding secondary nanoparticle pollution and process sustainability.

A critical bottleneck in modern water remediation is the “end-of-life” management of spent materials [17]. Traditional processes often result in the disposal of contaminated adsorbents and harvested pollutants, shifting the environmental burden from water to land and failing to align with the principles of green chemistry [18,19]. Consequently, increasing attention has been devoted to strategies that not only remove contaminants but also transform them into valuable resources, in line with the principles of the circular economy. The valorization of microplastics has recently emerged as a key research frontier within the transition toward a circular plastics economy, driven by the persistence, environmental mobility, and toxicity of these contaminants [20].

Among the different valorization routes proposed for PET waste, chemical depolymerization offers the possibility of recovering its original building blocks, terephthalic acid (TPA) and ethylene glycol (EG), which can be reused as chemical feedstocks [21]. Conventional methanolysis and glycolysis require organic solvents, catalysts, or high-pressure conditions, whereas acid- and alkali-catalyzed hydrolysis involves corrosive reagents and generates large amounts of inorganic salts during the neutralization step, increasing both environmental impact and downstream processing costs [22,23,24]. In contrast, neutral hydrolysis emerges as a highly selective and sustainable alternative. By utilizing subcritical water as the sole solvent and reactant, it entirely eliminates corrosion issues and saline byproducts [23]. Although overcoming the polymer’s hydrophobicity requires higher temperatures, the downstream separation is remarkably straightforward: upon cooling, the highly pure TPA naturally precipitates due to its aqueous insolubility, leaving the EG isolated in the liquid phase [25].

This recovered EG represents an attractive secondary raw material that can be directly reintroduced into new manufacturing processes, replacing virgin reagents and contributing to resource efficiency. In this work, EG recovered from PET hydrolysis is reused as the reaction medium for the synthesis of new MIONPs, establishing a closed-loop strategy in which the harvested MicroPET is transformed into functional nanomaterials for subsequent AOPs. These advanced catalytic systems [26], including magnetic induction- and solar-assisted AOPs [27,28], have demonstrated the ability to convert MicroPET into smaller organic molecules, fuels, or even energy carriers [29,30] under mild conditions. In this context, the synthesized MIONPs can actively drive these processes by exhibiting enzyme-like (nanozyme) activity. This capability enables heterogeneous Fenton-like reactions that generate highly reactive oxygen species (ROS), such as hydroxyl radicals (●OH) or hydroperoxyl radicals (●OOH), capable of oxidatively degrading target organic pollutants in aqueous systems [31].

In previous work, we demonstrated the efficient magnetic harvesting and degradation of microplastics from polyethylene extracted from cosmetics using NFs synthesized by a polyol-autoclave method [15]. Their multicore mesocrystalline structure promotes cooperative magnetic interactions between individual crystallites, resulting in enhanced magnetic response and improved performance during magnetic induction heating [32]. More recently, we successfully translated this synthesis procedure to a microwave-assisted polyol route, significantly reducing reaction times while increasing production yield [33], and presenting excellent performance of nanoplastics magnetic harvesting from water [34].

In the present work, we extend this concept by integrating MicroPET recovery, valorization, and reuse into a single circular process. MicroPET collected by magnetic separation is chemically depolymerized through neutral hydrolysis. The supernatant recovered is subsequently reused as the reaction medium for the synthesis of new magnetic iron oxide nanoparticles. The harvesting performance of the synthesized single-core (SC) nanoparticles and magnetic clusters (MCs) was evaluated under different operating conditions, including pH, particle-to-microplastic ratio, and contact time, and then compared with that of the original NFs. Finally, both pristine nanoparticles and MicroPET-loaded hybrid materials were assessed as heterogeneous Fenton-like catalysts for methylene blue (MB) decolorization under alternating magnetic field (AMF) assistance. This strategy demonstrates how recovered MicroPET can be transformed into new functional magnetic nanomaterials, establishing a circular route for water remediation that combines pollutant removal, material valorization, and catalytic reuse.

## 2. Results and Discussion

### 2.1. The Magnetic Carriers: NFs

The circular strategy proposed here starts from the magnetic carriers used to harvest MicroPET from water. These carriers are NFs prepared by the microwave-assisted polyol route previously reported by our group [33], using conventional diethylene glycol (DEG) and N-Methyldiethanolamine (NMDEA) as the reaction medium. Throughout this work, the NFs serve as the reference material against which the PET-derived nanoparticles described in Section 2.4, Section 2.5, Section 2.6 and Section 2.7 are compared. The complete structural, magnetic and magneto-thermal characterization of NFs is provided in Section S2 of the Supplementary Materials and is summarized below.

Figures S2 and S3 from Section S2 in the Supplementary Materials summarize the structural parameters and morphological characteristics of the NFs. High-resolution transmission electron microscopy (TEM, Figure S2A,B) analysis revealed a well-defined nanoflower morphology, with the samples exhibiting an average external diameter of 41 ± 5 nm and a primary core size of 20 ± 2 nm (Figure S2C). Additionally, a crystallite size of 36 nm was determined (Figure S2D). Consistent with previous reports [33], post-reaction sintering and partial coalescence drive this nanostructural evolution, yielding larger cores and aligned crystalline domains. This mesocrystal formation is confirmed by the HRTEM magnified image (Figure S2B), which reveals continuous lattice fringes extending across multiple cores.

Regarding magnetic performance, Figure S3A,B display the hysteresis loops recorded at 293 and 5 K, respectively. The NFs exhibit a superparamagnetic-like behavior at 290 K (M_s_ = 89 Am^2^ kg^−1^, M_R_/M_S_ = 0.01, and H_c_ = 0.7 kA m^−1^), due to thermally activated magnetization reversal [33]. Upon cooling to 5 K, the system shows a multidomain-like response (M_R_/M_S_ = 0.22, H_c_ = 15.8 kA m^−1^). The increase in low-temperature coercivity suggests changes in magnetic behavior associated with reduced thermal agitation. The enhanced magnetic anisotropy reflects contributions from intraparticle magnetic interactions, according to the structural organization within the mesocrystals.

Finally, Figure S3C illustrates the calorimetric heating curve of the NFs under an alternating magnetic field (200 kHz, 24 kA m^−1^), demonstrating a rapid temperature increase from 20 to 30 °C and highlighting their potential for magnetic hyperthermia applications.

### 2.2. The Target Pollutant: PET—MicroPET

**Figure 1:**
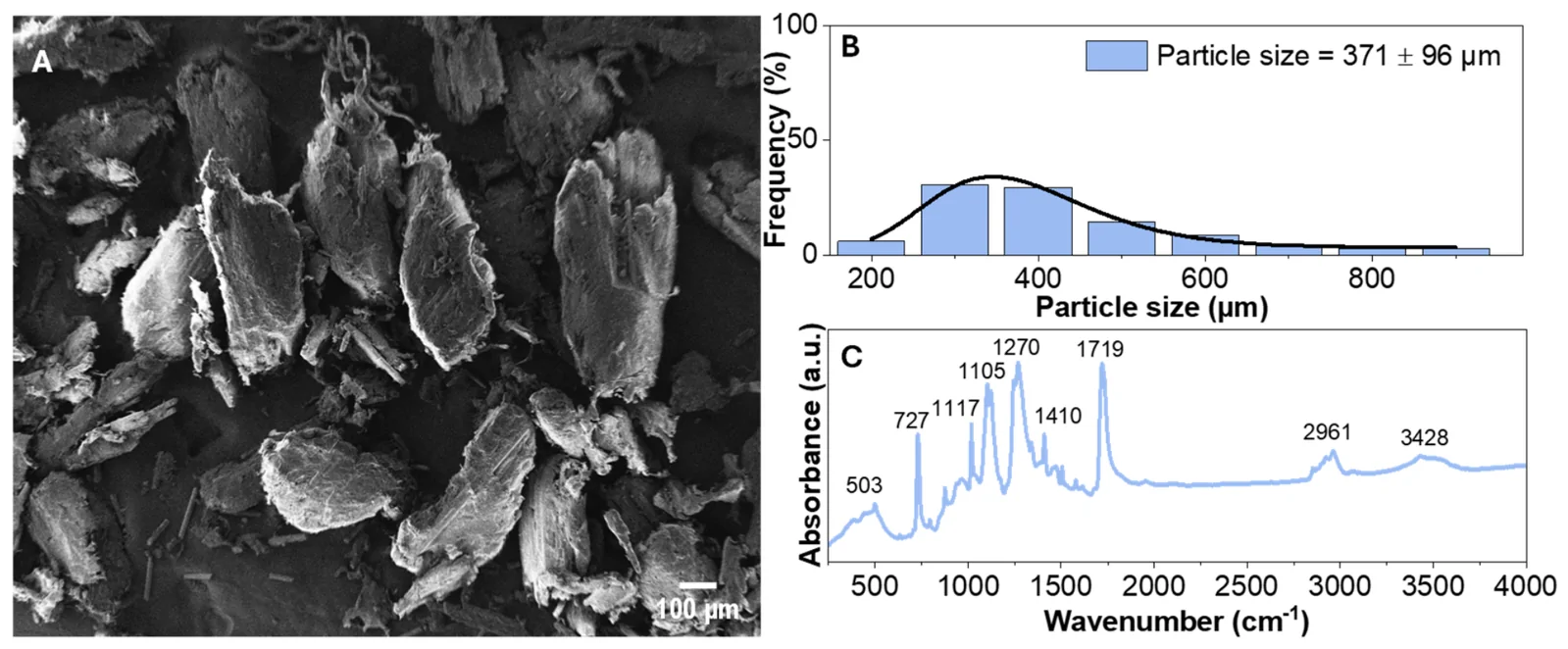
Morphological and physicochemical characterization of MicroPET. (**A**) SEM micrographs showing the fragmented and irregular morphology typical of MicroPET (scale bar: 100 μm). (**B**) Particle size distribution histogram showing a mean length of 371 ± 96 μm characteristic of the MicroPET fraction. (**C**) FTIR spectrum confirming the chemical identity of PET.

The surface morphology of MicroPET was analyzed using a scanning electron microscope (SEM), as shown in Figure 1A. The micrographs reveal that the material consists of irregular particles with elongated morphology and a rough surface texture, resembling scales or splintered fragments. Analysis of the particle size distribution (Figure 1B) shows an asymmetric distribution with a tendency toward larger sizes. The average particle length was determined to be 371 ± 96 μm, while the particle width was calculated to be 151 ± 63 μm (see Figure S4 of the Supplementary Materials).

To identify the functional groups, present in the material, an FTIR spectroscopy analysis was performed (Figure 1C). Consistent with the characteristic FTIR assignments reported for PET [34], the spectrum shows a broad band in the region of 3428 cm^−1^, attributable to O–H stretching vibrations, likely associated with adsorbed moisture or surface hydroxyl groups. The band observed at 2961 cm^−1^ corresponds to aliphatic C–H stretching vibrations. A sharp, intense signal at 1719 cm^−1^ stands out, characteristic of the stretching vibration of the carbonyl group (C=O), confirming the polyester nature of the sample. In the “fingerprint” region, the bands at 1270 cm^−1^ and 1105–1117 cm^−1^ are assigned to asymmetric and symmetric C–O–C stretching vibrations of the ester linkage, respectively. The peak at 1410 cm^−1^ is related to aromatic ring stretching, while the band at 727 cm^−1^ is attributed to out-of-plane bending of aromatic C–H bonds. The low-wavenumber signal at 503 cm^−1^ can be associated with skeletal vibrations of the aromatic ring. Overall, the spectrum is fully consistent with the characteristic vibrational signature of PET.

### 2.3. Magnetic Harvesting of MicroPET

**Figure 2:**
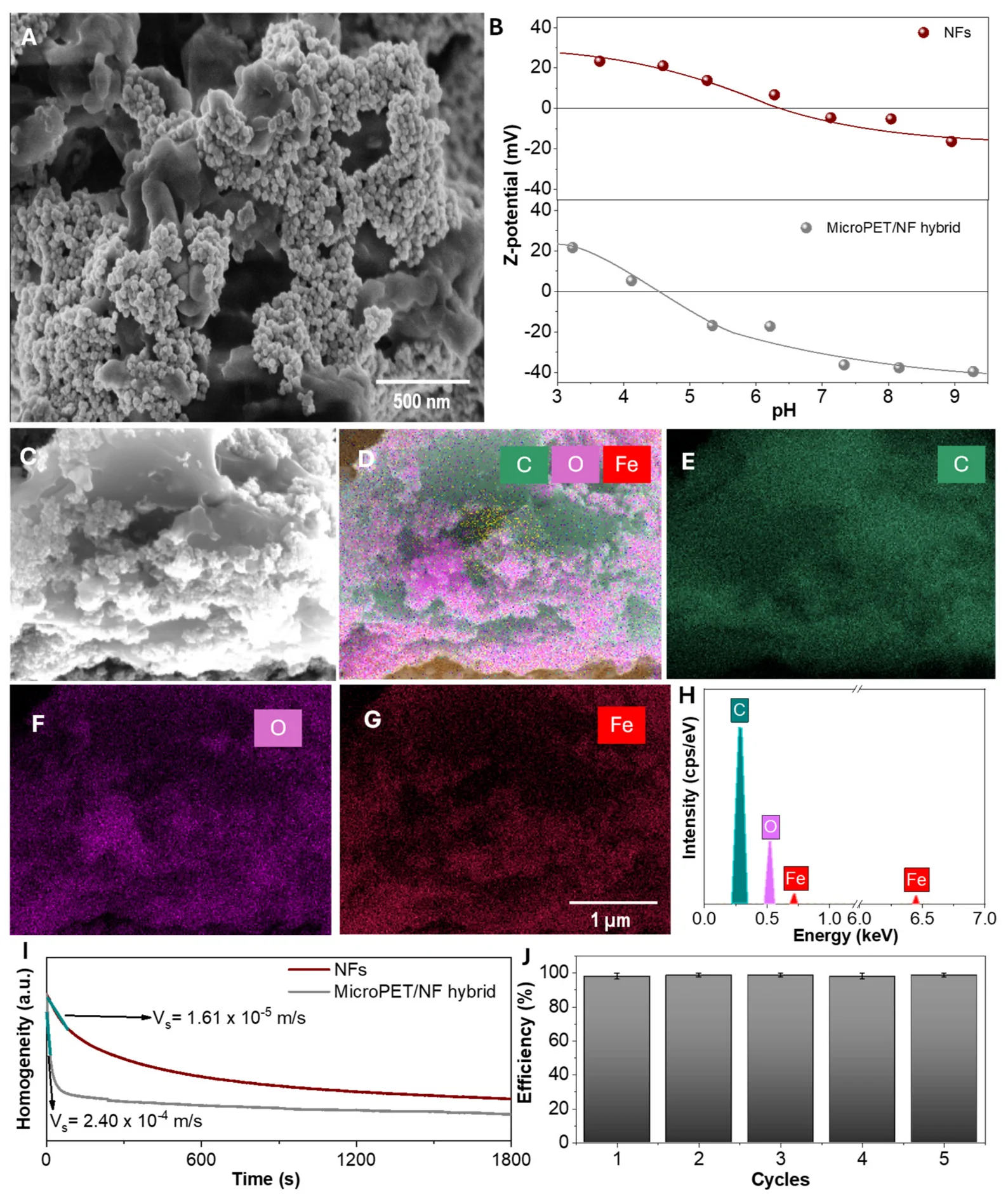
Morphological, physicochemical, and stability characterization of the MicroPET/NF hybrid. (**A**) SEM micrographs at different magnifications showing the morphology of MicroPET with surface-adhered NFs. (**B**) Zeta potential as a function of pH for pristine NFs and the hybrid MicroPET/NF, highlighting the shift in surface charge. (**C**–**G**) Reference SEM image and corresponding EDX elemental mapping illustrating the spatial distribution of carbon (C), oxygen (O), and iron (Fe). (**H**) EDX spectrum confirming the elemental composition of the hybrid. (**I**) Magnetic sedimentation kinetics (∇B = 24 T m^−1^) comparing the suspension stability of pristine NFs and the hybrid material over time. (**J**) Performance efficiency of the NFs evaluated over five consecutive reuse cycles, demonstrating high operational stability for magnetic harvesting.

Figure 2 illustrates the physical, chemical, and functional evaluation of the engineering MicroPET/NF hybrid system, consisting of MicroPET serving as a substrate for adhered NFs under optimal conditions (pH 7, 10 mg of NFs, 30 min of contact time) [15]. High-magnification SEM micrographs (Figure 2A) reveal that NFs maintain a characteristic hierarchical morphology, providing a high specific surface area that facilitates the dense attachment of MicroPET. From a physicochemical perspective, the zeta potential analysis (Figure 2B) provides critical evidence of this surface modification. These pristine NFs exhibit an isoelectric point (IEP) near pH 6.5, whereas the hybrid system shows a marked acidic shift, with the IEP dropping below 4.5 and presenting a significantly higher negative charge density at alkaline and neutral pH levels. This shift is primarily attributed to the presence of oxygenated functional groups on the MicroPET surfaces, which mask the native hydroxyl groups of iron oxide and fundamentally alter the particle-water interface.

The compositional integrity of this surface modification is further confirmed by SEM–Energy-Dispersive X-ray Spectroscopy (EDX) mapping (Figure 2C–H). The overlay (Figure 2D) and individual elemental maps (Figure 2E–G) demonstrate a homogeneous distribution of carbon (C), oxygen (O), and iron (Fe). The presence of a dominant carbon signal (Figure 2H), perfectly overlapping with the iron oxide core, qualitatively validates the surface loading of the MicroPET.

The surface changes documented in the zeta potential analysis directly dictate the colloidal stability of the system, as evaluated by the magnetic sedimentation kinetics (Figure 2I). Interestingly, although the hybrid material exhibits a highly negative zeta potential at a neutral pH, which would conventionally imply greater electrostatic repulsion and stability, it undergoes much more rapid settling compared to the pristine NFs. The hybrid system demonstrates settling velocity (V_s_) of 2.40 × 10^−4^ m s^−1^, an order of magnitude faster than the pristine NFs (1.61 × 10^−5^ m s^−1^). This accelerated sedimentation indicates that the physical properties of the heavier hybrid assembly (driven by the increased mass of the MicroPET/NF complex, hydrophobic interactions, or bridging flocculation between particles) dominate over electrostatic repulsion. This behavior is highly advantageous for practical applications, where rapid bridging flocculation acts as the driving force to accelerate magnetic sedimentation, thereby facilitating efficient material recovery from water.

To evaluate the reusability of the NF carriers, a targeted regeneration protocol was employed. In the initial stage, ethanol acts as a crucial wetting agent that reduces the surface tension of the aqueous medium, facilitating the individual dispersion of the inherently hydrophobic MicroPET and preventing their non-specific aggregation with the NFs which would otherwise compromise separation efficiency. Subsequently, the separation relies exclusively on a density gradient. A 65% sucrose solution (density ~1.3–1.5 g cm^−3^ at 25 °C) is uniquely suited to act as a barrier that is denser than water but less dense than PET (density 1.68 g cm^−3^ at 25 °C). This gradient precisely captures the MicroPET at the interface while allowing the significantly denser NFs to penetrate all layers; no other common density medium offers this combination of physical tuning, non-toxicity, high solubility for a clear interface, and cost-effectiveness essential for this processing [35]. Finally, the operational robustness of the hybrid is demonstrated in Figure 2J, where the recovered NFs retain nearly 100% of their initial recovery efficiency across five consecutive regeneration cycles.

### 2.4. Valorization of MicroPET as Raw Material for the Synthesis of MIONPs

This section demonstrates the successful use of the supernatant obtained from the neutral hydrolysis of PET as the reaction medium for the synthesis of MIONPs. MicroPET depolymerization was evaluated through two complementary approaches: (i) polymer conversion efficiency, determined under laboratory-scale conditions by gravimetric analysis of the hydrolyzed material, and (ii) monomer recovery, assessed in a reactor with a ten-fold larger working volume, where the generated TPA and EG were separated, recovered, and quantified.

To optimize the hydrolysis conditions, preliminary unscaled experiments were performed using 50 mg of MicroPET dispersed in 50 mL of water in a 100 mL reactor. The extent of depolymerization was evaluated gravimetrically as a function of temperature. As summarized in Figure S5 of Section S3 in Supplementary Materials, PET conversion increased markedly with increasing temperature, reaching only 10% at 150 °C, 70% at 200 °C, and 93.2% at 225 °C. The highest conversion was achieved at 250 °C, where a gravimetric PET conversion of 97% was obtained. Based on these results, 250 °C was selected as the optimal temperature for subsequent hydrolysis experiments.

#### 2.4.1. Spectroscopic Validation and Chemical Fingerprinting of the Recovered Synthesis Medium

Based on the optimal hydrolysis temperature identified in the unscaled experiments (250 °C, corresponding to a gravimetric PET conversion of 97%), the process was subsequently scaled up to a 1000 mL reactor using 30 g of MicroPET dispersed in 500 mL of water. This scale-up was intentionally performed to generate sufficient quantities of hydrolysis products for isolation, characterization, and subsequent nanoparticle synthesis, which would not have been feasible under the unscaled conditions. This section focuses on the identification and recovery of the products obtained from the scaled hydrolysis procedure.

**Figure 3:**
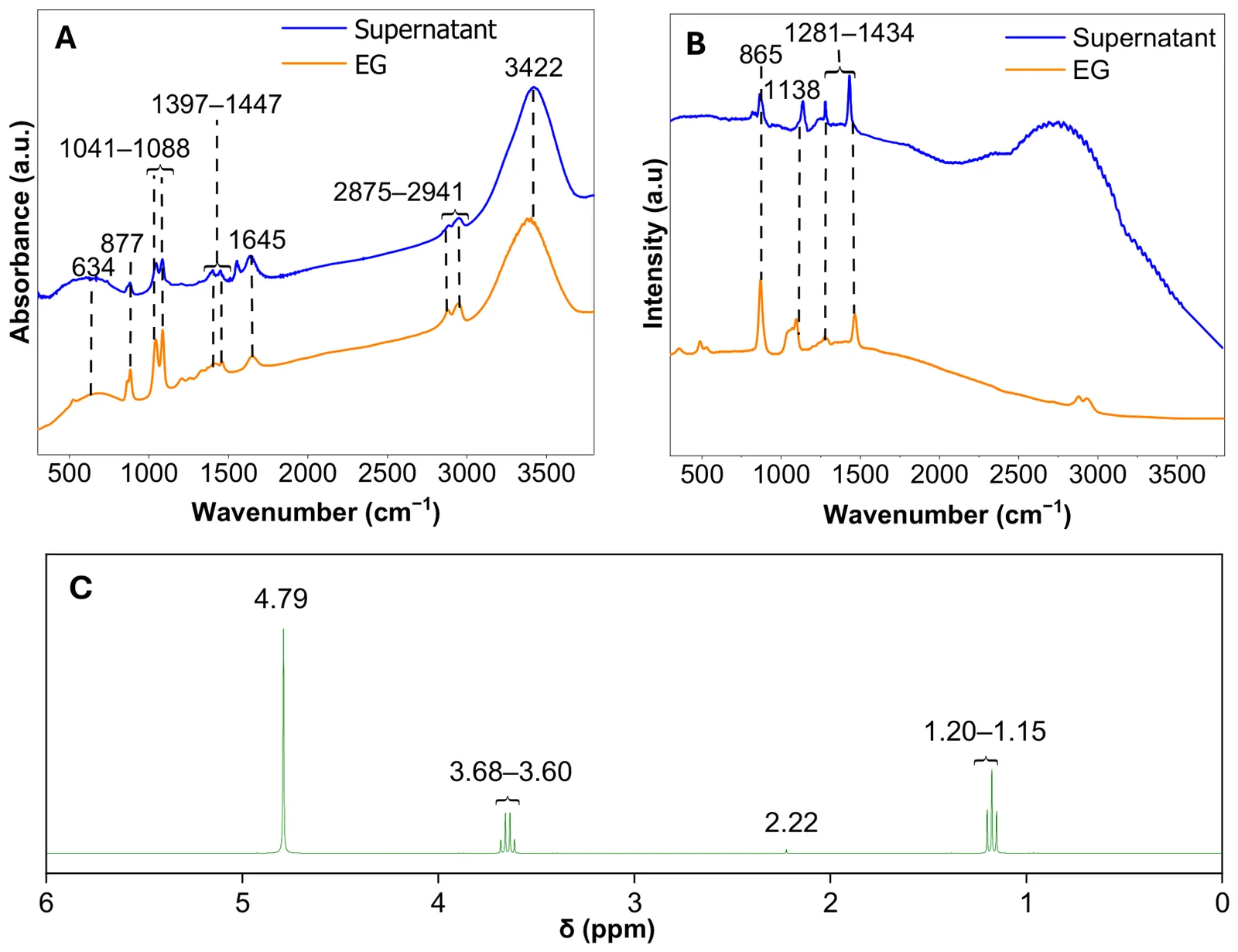
Spectroscopic characterization of the PET-derived supernatant and commercial EG. (**A**) FTIR absorbance spectra, (**B**) Raman spectra, and (**C**) ^1^H NMR spectra comparing the recovered supernatant. Additional characterization of the recovered TPA is provided in Figure S6.

The recovered products from the scaled procedure were identified as EG (Figure 3) and TPA (Section S4, Figure S6A,B) by spectroscopic analyses. Figure 3A,B compare the Fourier transform infrared (FTIR) and Raman spectra of the recovered supernatant (royal blue) with those of commercial EG (orange), confirming that the hydrolysis supernatant is predominantly composed of EG. The FTIR spectra exhibit the characteristic absorption bands of EG, including the broad O–H stretching vibration centered at 3422 cm^−1^, aliphatic C–H stretching vibrations between 2875 and 2941 cm^−1^, and the C–O stretching region at 1041–1088 cm^−1^. Additional bands assigned to O–H bending (1645 cm^−1^), C–H bending vibrations (1397–1447 cm^−1^), CH_2_ rocking (877 cm^−1^), and out-of-plane O–H bending (634 cm^−1^) are also observed in both samples [36,37]. The Raman spectra further support this assignment, showing the characteristic EG bands at 865, 1138, and 1281–1434 cm^−1^, corresponding to skeletal C–C and C–O vibrational modes [36,38]. The use of both spectroscopic techniques is highly complementary; while FTIR is sensitive to the dipole changes in polar functional groups such as hydroxyls and carbonyls, Raman spectroscopy excels at probing the non-polar carbon-backbone vibrations, together providing an unambiguous chemical fingerprint of the EG-rich supernatant [39].

To further establish the chemical identity of the recovered liquid phase and identify co-existing species, ^1^H-NMR spectroscopy was performed (Figure 3C). The spectrum was dominated by the characteristic resonances of EG centered at δ = 3.63–3.68 ppm. A strong signal at δ = 4.79 ppm was assigned to residual HDO in D_2_O. Additional low-intensity resonances were observed at δ = 2.22 ppm and δ = 1.15–1.20 ppm, which can be attributed to acetaldehyde and ethanol, respectively [40,41]. These results confirm the presence of EG as the predominant component of the recovered liquid fraction while also revealing the presence of minor volatile byproducts generated during the PET hydrolysis process.

Furthermore, to complement the spectroscopic identification with a quantitative mass balance, the recovered supernatant (2.5 mL) was analyzed by cryoscopic osmometry. To avoid exceeding the instrument’s measurement range, the sample was diluted 1:10 (*v*/*v*), yielding an osmolality of 1992 mOsm kg^−1^, equivalent to 1.992 mol kg^−1^ for EG, a non-electrolyte that does not dissociate in aqueous solution (Van’t Hoff factor, *i* = 1) [42]. After correcting for the dilution factor, the recovered supernatant contained 0.01793 mol mL^−1^ of EG, corresponding to 1.113 g mL^−1^ based on its molar mass (62.07 g mol^−1^). This value is in excellent agreement with the reported density of pure EG at room temperature. Together with the FTIR and Raman analyses, these results confirm the successful recovery of EG following PET neutral hydrolysis.

Based on the recovered volume (2.5 mL), approximately 2.78 g (~2.49 mL) of EG was obtained. Complete depolymerization of 30 g of PET theoretically yields 25.93 g of TPA and 9.69 g (~8.68 mL) of EG. While the unscaled hydrolysis experiments showed a PET conversion of approximately 97% based on gravimetric analysis, the scaled hydrolysis resulted in the recovery of 7.44 g of purified TPA and 2.78 g of EG, corresponding to isolated yields of 28.7% of the theoretical values for each product (Table 1).

The discrepancy between the conversion achieved under unscaled conditions and the isolated yields obtained during scale-up is attributed to several factors. First, the unscaled experiments were performed using only 50 mg of PET in 50 mL of water within a 100 mL reactor, maximizing polymer accessibility and favoring efficient hydrolysis, which resulted in a gravimetric conversion of 97%. In contrast, the scaled experiments involved 30 g of PET dispersed in 500 mL of water inside a 1000 mL reactor. This scale-up was intentionally performed to generate sufficient quantities of hydrolysis products for isolation, characterization, and subsequent nanoparticle synthesis, which would not have been feasible using the small quantities produced under unscaled conditions. Under these unstirred conditions, the significantly larger polymer loading promoted the formation of compact PET aggregates that hindered water penetration and reduced depolymerization efficiency. Furthermore, the isolated yields reflect recovery efficiency rather than actual PET conversion, with additional losses arising from the partial solubility of TPA in the mother liquor, retention of EG in the aqueous phase, the presence of unrecovered soluble oligomers, and mechanical losses during filtration, washing, and product transfer. Therefore, while the unscaled experiments demonstrate that near-complete PET depolymerization can be achieved, the scaled process was primarily designed to produce sufficient quantities of recovered products for downstream valorization rather than to maximize monomer recovery yields.

Although previous studies have reported reduced EG recovery due to co-evaporation or thermal degradation during processing [43,44], the present protocol enabled the simultaneous recovery of both depolymerization products with minimal additional losses. Overall, the combined spectroscopic and osmometric analyses validate the recovery protocol and demonstrate that the recovered EG is suitable for reuse as the solvent in the subsequent synthesis of magnetic nanoparticles.

#### 2.4.2. PET-Derived MIONPs

**Figure 4:**
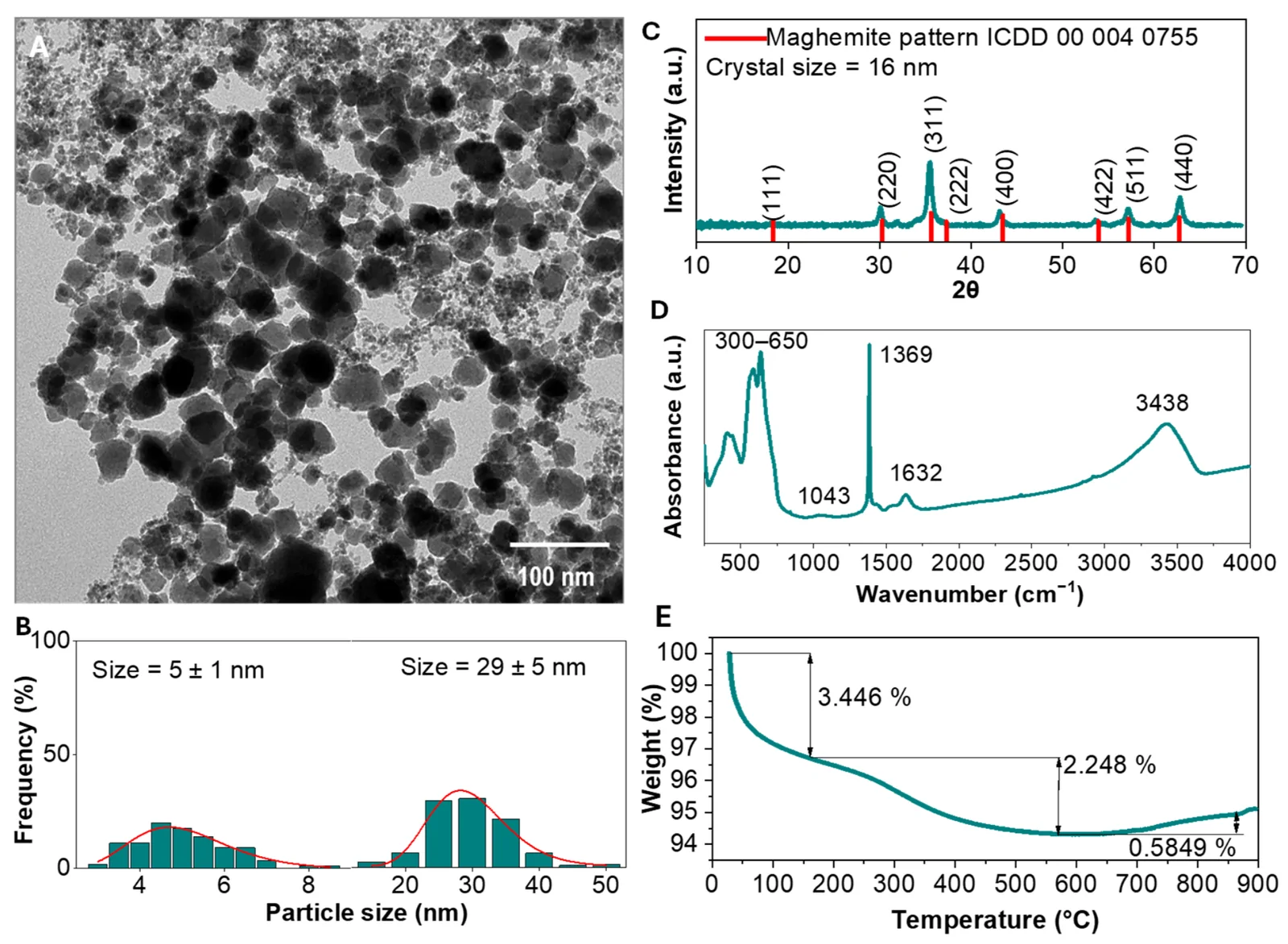
Characterization of the primary nanoparticles (SC): (**A**) TEM micrograph, (**B**) bimodal particle size distribution histogram (red line correspond to the lognormal fitting), (**C**) X-ray diffraction pattern, (**D**) FTIR spectrum and (**E**) TG analysis.

Having established the chemical identity of the recovered liquid fraction, the MicroPET-derived EG was then evaluated as a reaction medium for the synthesis of magnetic iron oxide nanoparticles, thereby closing the material loop of the proposed strategy. To obtain these nanoparticles, the microwave-assisted synthesis method originally designed for engineering NFs was adapted by substituting the conventional reaction medium DEG and NMDEA [33] with the PET-derived supernatant, containing EG and TPA. Following the synthesis, Figure 4A–E characterize the resulting primary SC. The TEM image (Figure 4A) and size distribution analysis (Figure 4B) reveal a bimodal population consisting of small single-core particles (5 ± 1 nm) and larger crystalline structures (29 ± 5 nm). The XRD pattern (Figure 4C) confirms that these particles consist of maghemite (γ-Fe_2_O_3_, ICDD 00-004-0755) with an average crystallite size of 16 nm. The surface chemistry and organic coating of the SC were detailed via FTIR and thermal gravimetric analysis (TGA). The FTIR spectrum (Figure 4D) confirms the formation of an iron oxide phase through the characteristic Fe–O lattice vibrations observed below 700 cm^−1^. Notably, both Fe–O bands exhibit a clear splitting, a spectral feature commonly associated with maghemite (γ-Fe_2_O_3_) rather than magnetite [45]. This observation is consistent with the oxidative acid treatment applied after synthesis, which was deliberately introduced to ensure the complete oxidation of magnetite into maghemite. The band at 1369 cm^−1^ is assigned to residual nitrate species originating from the nitric acid/iron nitrate oxidizing medium. In addition, the band at 1043 cm^−1^ is assigned to C–O stretching vibrations from residual EG, while the peak at 1632 cm^−1^ is attributed to overlapping contributions from adsorbed water bending vibrations and oxygen-containing surface species. The broad band centered at 3438 cm^−1^ is attributed to O–H stretching vibrations from surface hydroxyl groups and adsorbed water molecules [39]. This interpretation is complemented by the TGA thermograms (Figure 4E), which reveal a total mass loss of approximately 3 wt.%. The initial weight loss below 100 °C is attributed to adsorbed water and surface hydroxyl groups, whereas the mass loss observed between 150 and 600 °C is associated with residual organic matter on the nanoparticle surface. Considering that EG was the only organic reagent employed during the synthesis, it is expected to represent the major contribution to this organic fraction, although the presence of minor PET-derived species cannot be completely excluded.

The structural deviation from the well-ordered “nanoflower” morphology to a bimodal single-core architecture highlights a fundamental technical and environmental compromise inherent to this green synthesis strategy. In the traditional protocol, the NMDEA/DEG system serves as a precise structural director; NMDEA acts as a buffering and coordinating agent that controls the nucleation rate and stabilizes primary cores, facilitating their oriented aggregation into highly anisotropic mesocrystals [32,33]. Conversely, replacing this system with the EG/TPA supernatant suppresses this directed assembly pathway. Mechanistically, changing the polyol from DEG to EG alters the solvent viscosity and reducing power, while the absence of NMDEA deprives the system of the specific amine-driven capping needed to align the primary nuclei. Consequently, advanced magnetic features, such as collective spin behavior, high magnetic anisotropy, and optimized SAR for magnetic hyperthermia, are sacrificed.

Despite these structural and magnetic differences, the use of the recovered hydrolysis products represents a significant environmental advantage by replacing virgin organic reagents with a secondary feedstock derived from PET waste, reducing both the environmental impact and the cost associated with nanoparticle synthesis. More importantly, as demonstrated in the following sections, these changes do not substantially compromise the practical performance of the nanoparticles. The materials retain excellent magnetic harvesting efficiency toward MicroPET and remain effective heterogeneous Fenton-like catalysts for MB decolorization under alternating magnetic field assistance. Therefore, the recovered EG provides a viable reaction medium for producing functional maghemite nanoparticles while closing the material loop through the valorization of PET waste.

Beyond the SC described above, the same PET-derived medium also yielded a second architecture when the iron precursor and the thermal profile were modified (Section S1 of the Supplementary Materials): magnetic clusters (MCs), consisting of secondary aggregates of ~60 ± 14 nm assembled from ~10 ± 5 nm maghemite nanocrystals. XRD confirms that the maghemite phase is retained, while TGA reveals a markedly higher organic content than the SC counterpart (>39 wt.% vs. ~3 wt.%), attributed to the coordination of PET-derived species (mainly residual TPA) during synthesis. The complete structural, physicochemical, magnetic harvesting characterization of the MCs is provided in Sections S5–S8 (Figures S7–S10 and Table S1); their magnetic and catalytic behavior is compared with that of the SC at the corresponding points below.

### 2.5. Hybridization of MicroPET with SC by Magnetic Harvesting

**Figure 5:**
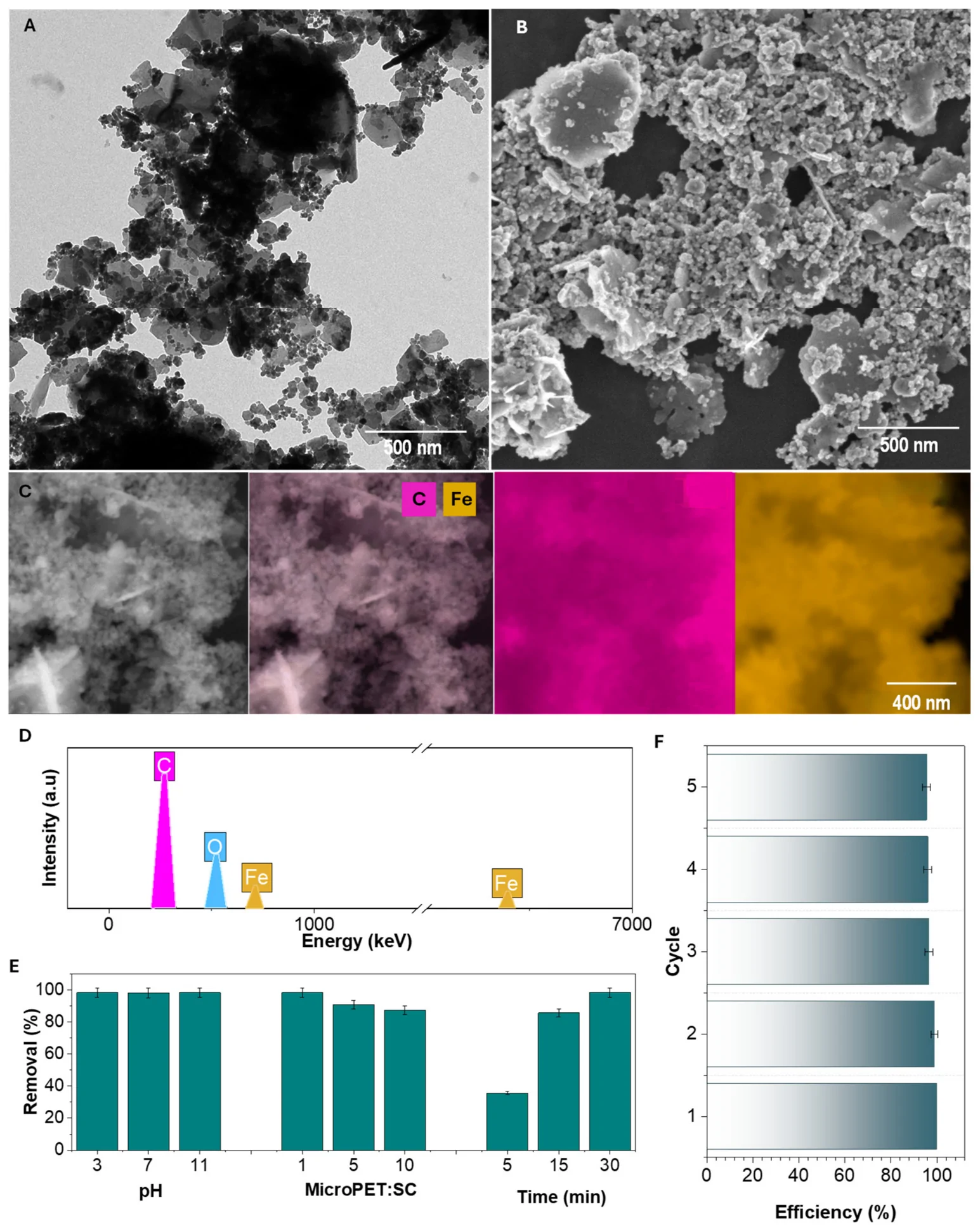
Characterization and operational optimization of the MicroPET/SC hybrid assemblies. (**A**,**B**) TEM and SEM images revealing tight interfacial connection and uniform distribution of SC across the irregular MicroPET matrix. (**C**) Elemental mapping demonstrates the spatial co-localization of C and Fe across the hybrid architecture. (**D**) EDX spectrum confirming the elemental composition. (**E**) Effect of initial pH, MicroPET/SC mass ratio, and contact time on the removal efficiency, showing optimized conditions for the ultrasound-assisted interfacial association process. (**F**) Performance efficiency of the SC evaluated over five consecutive reuse cycles. Control experiments performed in the absence of SC nanoparticles confirmed negligible MicroPET magnetic removal, demonstrating that the observed harvesting results from the formation of MicroPET/SC hybrid assemblies rather than spontaneous sedimentation or precipitation. Magnetic harvesting experiments were performed by triplicate while reusability assays by duplicate.

The integration of MicroPET with SC was achieved through an ultrasound-assisted interfacial association process followed by magnetic separation. As shown in Figure 5A (TEM) and Figure 5B (SEM), the SC are uniformly distributed over the irregular MicroPET surface, forming stable hybrid assemblies rather than isolated magnetic aggregates. This intimate polymer–oxide interaction is further confirmed by the elemental mapping analysis (Figure 5C,D), which reveals the homogeneous co-localization of Fe and C throughout the hybrid structure, demonstrating effective interfacial adhesion.

The formation of the hybrid assemblies was mainly governed by the contact time and the MicroPET-to-SC mass ratio, whereas the solution pH had only a minor influence (Figure 5E). Increasing the amount of MicroPET promoted progressive surface coverage until saturation, while longer incubation times improved the stability of the hybrid assemblies prior to magnetic separation. Under the optimized conditions (neutral pH, 30 min contact time, and a 1:1 mass ratio corresponding to 10 mg of each component in 2 mL), a maximum harvesting capacity of 1000 mg of MicroPET per gram of SC was achieved.

Although only a limited pH dependence was observed, other environmental factors may influence nanoparticle–MicroPET interactions under real conditions. For example, variations in ionic strength, dissolved organic matter, or competing suspended particles could modify the frequency and strength of particle–particle interactions. Likewise, environmental weathering of PET can increase surface roughness and introduce oxygen-containing functional groups, potentially altering its affinity toward iron oxide nanoparticles. While these effects were beyond the scope of the present proof-of-concept study, they may influence harvesting performance in natural waters and warrant further investigation.

Interestingly, this harvesting capacity is identical to that previously reported for magnetic NFs under the same operating conditions [15], despite the lower magnetic response of the SC resulting from the absence of the multicore mesocrystalline architecture. At first glance, a lower magnetic response would be expected to slow down magnetic collection. However, for MicroPET particles with characteristic sizes of several hundred micrometers, the separation process is primarily governed by the formation of large hydrophobic hybrid assemblies rather than by the intrinsic magnetic properties of the nanoparticles. Consequently, the reduced magnetic performance of the SC does not translate into a measurable loss in harvesting efficiency under these conditions.

This behavior contrasts with the recovery of NanoPET, where the much smaller particle size substantially increases the surface area to be covered and requires greater magnetic mobility of the carrier particles. In our previous work, the multicore NFs achieved harvesting capacities of up to 10,000 mg g^−1^ for NanoPET [34], highlighting the advantage of their enhanced magnetic properties for the capture of nanoscale plastic contaminants.

To evaluate the operational stability of the PET-derived SC nanoparticles, the recovered materials were subjected to five consecutive MicroPET harvesting cycles. As shown in Figure 5F, the harvesting efficiency exhibited only a slight decrease from 98.9% in the first cycle to 95.5% after the fifth cycle. This minor reduction is attributed to the regeneration procedure, in which the MicroPET/SC hybrids were separated using a sucrose-density gradient followed by multiple washing and magnetic recovery steps. These successive processing stages may lead to small nanoparticle losses and incomplete detachment of MicroPET fragments from the particle surface, progressively reducing the number of available active sites for hybrid formation.

Although this behavior differs from that previously observed for the NFs, which maintained nearly constant harvesting efficiencies over repeated cycles (Figure 2J), it is important to consider the structural differences between both systems. Nevertheless, despite this structural simplification, the PET-derived SC nanoparticles retained more than 95% harvesting efficiency after five consecutive cycles, presenting the additional advantage of being synthesized from waste-derived feedstocks.

Furthermore, the performance of MicroPET/SC hybrid and its maximum harvesting capacity were compared with alternative magnetic carriers reported in the literature, focusing on the relationship between the target MicroPET particle size and the corresponding harvesting ratio. As presented in Table 2, the MIONPs developed in this study achieved an exceptional maximum harvesting ratio of 1000 mg g^−1^ for the removal of MicroPET particles with a mean size of 370 μm.

This performance represents a major advancement over other advanced magnetic formulations and standard iron oxide carriers targeting similar plastic dimensions. For example, when dealing with MicroPET of comparable intermediate dimensions, such as the ≈200 μm particles targeted by Nano-Fe@ZIF-8 [50] or the ≈80 μm particles addressed by conventional Fe_3_O_4_ NPs [48], the literature reports substantially lower harvesting ratios of 25–165 mg g^−1^ and 67 mg g^−1^, respectively. Similarly, even when utilizing specialized morphological architectures like 451.1 nm Fe_3_O_4_ nanodisks to target much finer microplastic fractions (10 μm), the system yields a limited harvesting ratio of 188.4 mg g^−1^ [46]. This prominent discrepancy indicates that the SC matrix offers a vastly superior density of active surface sites or stronger interfacial binding affinities for mid-range MicroPET than metal–organic framework composites or unmodified nano-oxides. Furthermore, although an identical harvesting ratio of 1000 mg g^−1^ has been recorded in previous studies using iron oxide nanoparticles [49], those values were restricted to either significantly smaller microplastic fractions (38–150 μm) or much larger macro-sized distributions (1000–8000 μm), leaving a clear performance gap in the intermediate size regime that this work effectively addresses. Crucially, while references [47,49] and the aforementioned Fe_3_O_4_ nanodisc benchmark [46] rely on Fe_3_O_4_, a mineral phase highly prone to environmental oxidation and gradual loss of magnetic responsiveness, the deployment of a stable maghemite phase in this work guarantees long-term chemical durability under ambient conditions while successfully maintaining the absolute benchmark capacity of 1000 mg g^−1^. Therefore, the proposed system demonstrates an idealized combination of high chemical stability and maximum extraction efficiency specifically optimized for the remediation of intermediate 370 μm MicroPET contaminants.

Interestingly, even higher harvesting capacities have been previously reported by our group for PET nanoplastics (~150 nm) using NFs, reaching a record value of 10,000 mg g^−1^ [34]. This one-order-of-magnitude increase compared to the present MicroPET system highlights the strong influence of plastic size on magnetic harvesting performance. Due to their substantially lower mass, nanoplastic particles require fewer attached magnetic carriers to become magnetically recoverable, enabling significantly higher harvesting capacities than those attainable for larger microplastic fractions.

### 2.6. Magnetic Properties and Hyperthermia

**Figure 6:**
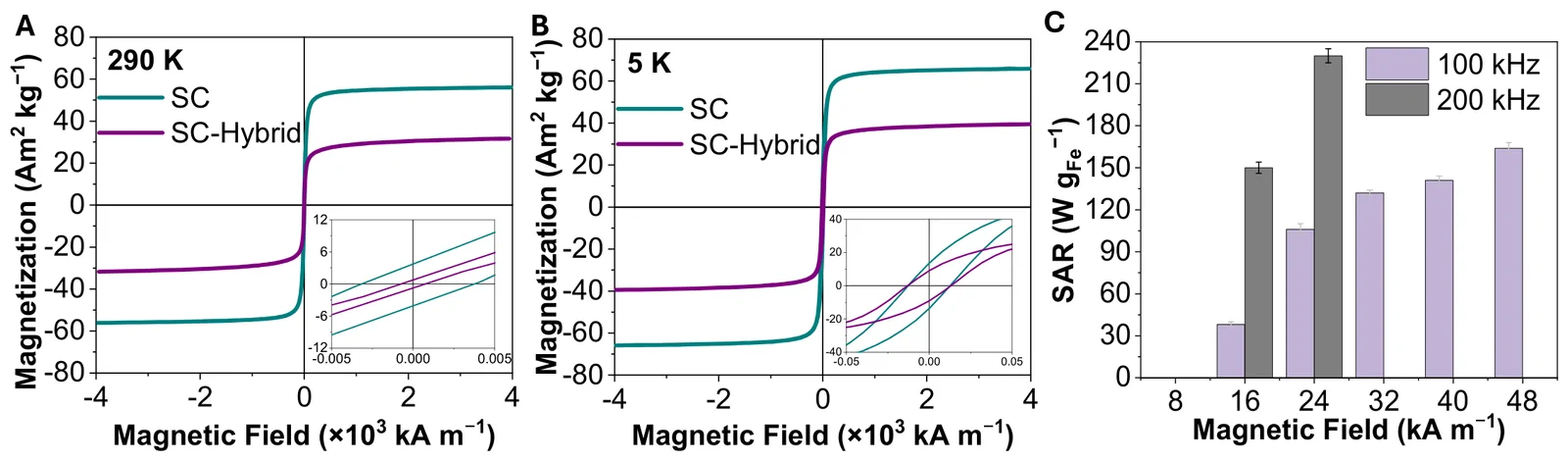
Magnetic properties and SAR performance for pristine SC and the MicroPET/SC hybrid. (**A**,**B**) Hysteresis loops (magnetization vs. applied magnetic field) at (**A**) 290 K, exhibiting superparamagnetic-like behavior with negligible H_c_ and remanence, and (**B**) 5 K, showing blocked state behavior. Insets display magnified views of the low-field regions. The reduction in saturation magnetization (M_S_) in the hybrid sample is attributed to the presence of the nonmagnetic MicroPET coating. (**C**) SAR values normalized per gram of iron for pristine SC as a function of the applied magnetic field at frequencies of 100 kHz and 200 kHz.

Figure 6A,B display the hysteresis loops (magnetization vs. applied magnetic field), revealing that at room temperature (290 K), all samples exhibit superparamagnetic-like behavior characterized by practically negligible coercivity (H_c_) and remanence (M_r_), with the saturation magnetization (M_s_) following the order SC > SC Hybrid. The extracted magnetic parameters M_s,_ M_r_ and H_c_ are summarized in Table 3 and compared with the magnetic properties of NFs. NFs exhibit superior magnetic performance across all conditions, recording the highest saturation magnetization of 89 Am^2^ kg^−1^ at 290 K and 98 Am^2^ kg^−1^ at 5 K well above the values obtained for the SC and the hybrid assemblies (57 and 67 Am^2^ kg^−1^ at RT and 5K, respectively). This outstanding magnetic response contrasts favorably with similar nanostructures reported in the literature [51]; for instance, single-core nanoparticles (NP15) previously reported achieved lower saturation magnetization of 78 Am^2^ kg^−1^, respectively. At 290 K, the SC and MicroPET/SC samples display characteristic superparamagnetic-like behavior with coercivity values from 0.7 to 3.4 kA m^−1^ and dimensionless remanence ratios from 0.7 to 3.8. These values are in excellent agreement with the superparamagnetic-like response of NP15 (H_c_ = 2.0 kA m^−1^). Conversely, the NFs lead to a distinctively higher H_c_ (0.7 kA m^−1^) at 290 K. Cooling the system to 5 K effectively blocks the particles, triggering a marked increase in M_s_, H_c_ and M_r_ for all samples. This magnetically blocked state is evidenced by the increases in the H_c_, reaching 15.8 kA m^−1^ for the NFs and 12 kA m^−1^ for the SC and hybrid systems, along with an increase in the M_r_/M_s_ ratio to values between 0.20 and 0.24. Nonetheless, these dimensionless remanence ratios remain below the theoretical threshold of 0.5 expected for randomly oriented, non-interacting single domain particles [52]. As anticipated, M_S_ undergoes a substantial decrease upon hybrid formation (dropping to 33 Am^2^ kg^−1^ at 290 K) due to the presence of nonmagnetic organic material (MicroPET).

Finally, the structural and magnetic advantages of the NFs are also reflected in their magneto-thermal efficiency, achieving superior SAR values (710 W g _Fe_^−1^ at 200 kHz and 24 kA m^−1^) compared to the isolated SC systems (230 W g _Fe_^−1^ at 200 kHz and 24 kA m^−1^). The measured SAR values align closely with literature data for NFs prepared via conventional autoclave routes, validating the successful adaptation of this synthesis to a microwave-assisted methodology [32]. Furthermore, these findings correlate well with the superior thermal dissipation anticipated by structurally well-defined NFs.

Figure 6C presents the SAR normalized per gram of iron (W g _Fe_^−1^) as a function of the applied magnetic field for the pristine SC. At a frequency of 200 kHz, the SC samples show a consistently higher SAR value than at 100 kHz (230 vs. 164 W g _Fe_^−1^) using a magnetic field of 24 kA m^−1^. This suggests that at this frequency, relaxation mechanisms are optimized for isolated single cores. Our results are comparable with SAR values from similar systems in the literature under comparable testing conditions [51,53].

### 2.7. Application of MicroPET/MION Hybrids

**Figure 7:**
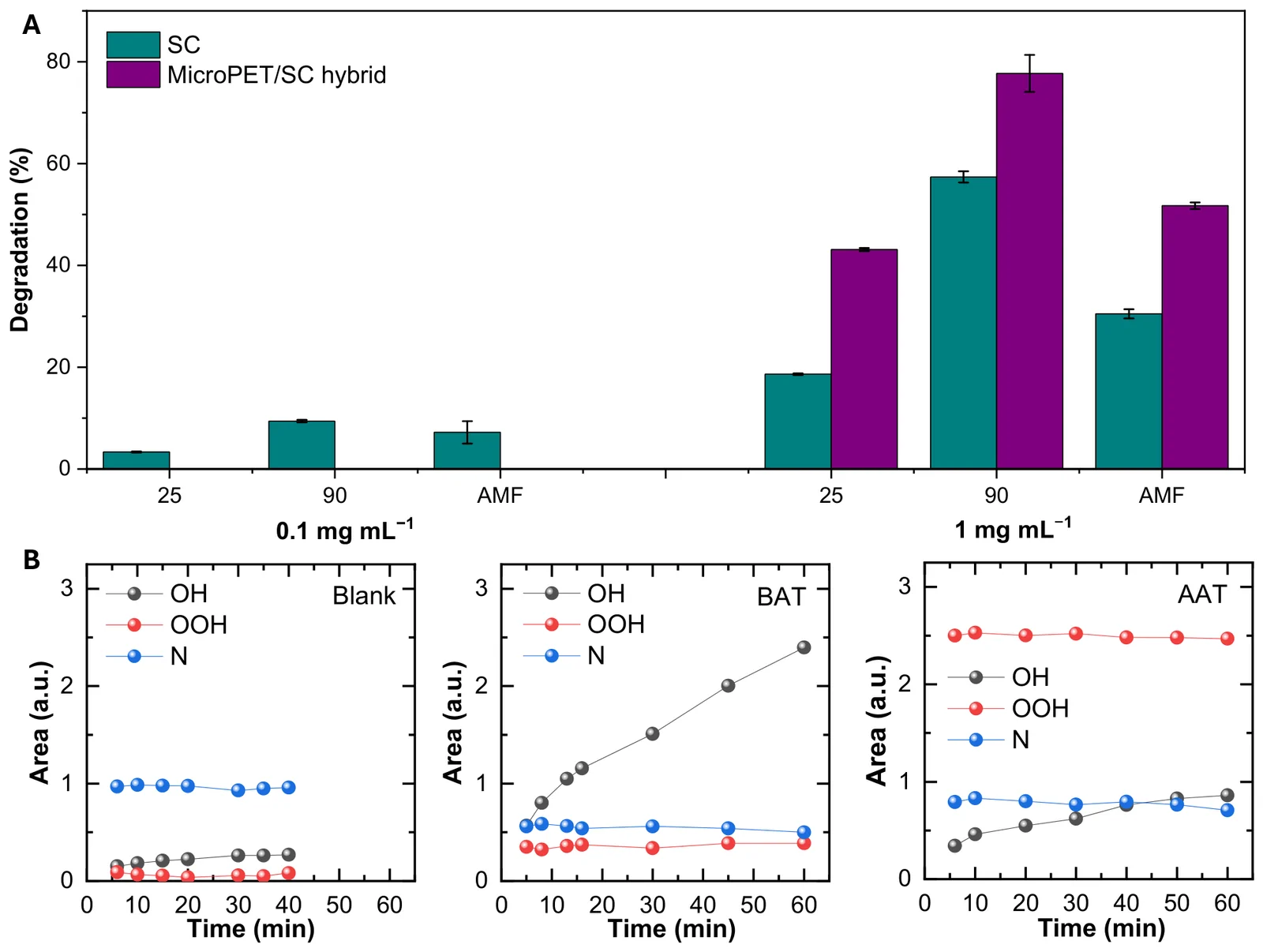
(**A**) Decolorization efficiency of MB degradation via a heterogeneous Fenton-like reaction activated with H_2_O_2_ (100 µL). The bar chart compares the catalytic performance of pristine SC and the MicroPET/SC hybrid at catalyst dosages of 0.1 mg mL^−1^ and 1.0 mg mL^−1^ under three distinct conditions: at 25 °C, 90 °C, and AMF; 200 kHz, 24 kA m^−1^ stimulation. [MB]_0_ = 100 ppm, reaction time = 2 h. Degradation assays started after 2 h of preconcentration leading to adsorption equilibria and were performed by triplicate. (**B**) Time evolution of the DMPO-adduct resonance areas (OH, OOH and N) for the blank (left), the as-synthesized SC sample before acidic treatment (BAT, center), and after acidic treatment (AAT, right).

Figure 7 illustrates the decolorization efficiency of MB degradation via a heterogeneous Fenton-like reaction activated with H_2_O_2_ using the MicroPET/SC hybrid. At a catalyst concentration of 0.1 mg ml^−1^, decolorization is negligible (<10%) under any stimulus, but upon increasing the dose to 1 mg ml^−1^ the decolorization of MB increases, underscoring the need for a critical density of active sites to generate sufficient free radicals. The pristine SC and MicroPET/SC hybrid show a low decolorization at 25 °C (~20–40%) and high decolorization under conventional heating at 90 °C (~57–78%), as thermal energy easily overcomes the energy activation of the Fenton reaction. Under magnetic induction (AMF: 200 kHz, 24 kA m^−1^), the decolorization is increased by ~20% compared to the RT conditions when using the MicroPET/SC hybrid. These findings imply that enhanced catalytic activity does not stem from bulk thermal increases within the reaction medium; instead, it points toward localized phenomena driven by the magnetic excitation of the hybrid assemblies. Given that all trials were conducted under constant agitation, the contribution of mechanisms restricted to macroscopic mass transfer alterations or continuous field-driven particle agglomeration is expected to be negligible.

Moreover, this optimization is directly linked to adsorptive behavior, as the integration of MicroPET elevates the local density of MB molecules near the active iron catalytic centers. Prior to the AMF-assisted degradation experiments, the MicroPET/SC hybrids were allowed to reach adsorption equilibrium with MB for 2 h, ensuring that the subsequent removal originated from catalytic degradation rather than from ongoing adsorption. Furthermore, we previously demonstrated that adsorption is a critical prerequisite for efficient degradation, as suppressing contaminant adsorption onto the catalyst surface resulted in a drastic reduction in degradation efficiency, highlighting the central role of surface preconcentration in heterogeneous oxidation processes [54]. Within heterogeneous Fenton frameworks, the surface preconcentration of contaminants reinforces the synergy between adsorption and catalytic oxidation stages, thereby boosting the likelihood that locally generated reactive oxygen species interact with target species prior to migrating into the bulk phase [54,55,56].

This adsorption–degradation coupling is consistent with our previous work on NanoPET/NF hybrids, where the adsorption of MB onto PET-containing magnetic composites was systematically evaluated prior to AMF-assisted degradation experiments. Consequently, incorporating MicroPET consolidates the adsorption phase of the catalytic pathway, leading to an upgraded overall reaction efficiency under both ambient conditions and AMF stimulation.

In addition, to determine which specific radical species is responsible for MB degradation, the free radicals generated by the pristine SC nanoparticles were identified by electron paramagnetic resonance (EPR) spectroscopy using DMPO as a spin trap. Figure S11 shows representative EPR spectra of the blank solution, and of the reaction solutions containing the SC sample, both before and after the acidic treatment. Comparison of the spectral features with the NIEHS Spin-Trap database [57] allowed identification of the DMPO-N adduct characterized by three equidistant lines and the DMPO-OH adduct characterized by four lines with a 1:2:2:1 intensity ratio in the as-made sample. In contrast, the spectrum of the acid-treated sample is dominated by the DMPO-OOH adduct. The experimental spectra were fitted using the hyperfine coupling constants reported for each adduct, as shown in Figure S11, and the resonance area of each species was followed as a function of reaction time, as presented in Figure 7B.

The DMPO-N adduct was detected in all the samples, with similar intensity, and showed no significant dependence on reaction time, indicating that its origin is not related to the catalytic activity of the nanoparticles, consistent with previous reports [58] attributing this signal to a nitrone-related species not resulting from the Fenton reaction itself [58,59,60]. Accordingly, the N adduct was excluded from the analysis.

For the SC as-synthetized sample, the ●OH radical was the predominant detected species. Its resonance area increased approximately linearly over the whole measured interval and its magnitude is higher than the observed blank, while the ●OOH contribution remains comparatively low and essentially constant. This behavior is consistent with the classical Fenton reaction (Fe^2+^ + H_2_O_2_ → Fe^3+^ + •OH + OH−), indicating that a significant proportion of Fe^2+^ remains after the synthesis and is accessible at the nanoparticle surface contributing to the catalytic activity.

After the acidic treatment, the DMPO-OOH adduct became the predominant radical species and remains detectable throughout the measurement period. This response is consistent with the Fenton-like reaction involving Fe^3+^ species (Fe^3+^ + H_2_O_2_ → Fe^2+^ + •OOH + H^+^), as expected for an oxidized maghemite sample.

In performance terms, the MCs behave analogously to the SC: they reach the same maximum harvesting capacity (1000 mg MicroPET g^−1^, Section S6, Figure S8) and reproduce the same AMF-assisted Fenton-like decolorization trend (Section S8, Figure S10). Both PET-derived architectures are therefore equally suitable for the circular water remediation scheme proposed here.

The applicability of AMF-assisted catalytic processes extends well beyond the degradation of methylene blue. As summarized in Table S2 in Section S9 of the Supplementary Materials, magnetic-field-assisted Fenton and Fenton-like systems have been successfully applied to structurally diverse contaminants, including antibiotics (tetracycline) [61], endocrine-disrupting compounds (bisphenol A) [62], cationic and anionic dyes (methylene blue [34,51,63], Acid Orange 8 [51], and methyl orange [64]), as well as complex environmental matrices such as textile wastewater and landfill leachate [64]. Several of these examples originate from our previous studies on magnetically induced heterogeneous Fenton catalysis, where iron oxide-based nanocatalysts operating under AMF stimulation achieved decolorization efficiencies ranging from 40 to 100% for model dyes [34,51,63] and mineralization efficiencies up to 74.7% for highly complex matrices like landfill leachate [64] or 80% for polyethylene microplastics from cosmetics [15]. More recently, the same approach was successfully extended to the degradation of polyethylene microplastics, further demonstrating the versatility of magnetically activated advanced oxidation processes [15].

Within this context, the MicroPET/SC hybrid developed in the present work achieved 51.7% methylene blue decolorization under AMF stimulation. Despite being assembled from recovered MicroPET and SC nanoparticles synthesized using PET-derived hydrolysis products, the hybrid exhibited catalytic performances comparable to those previously reported for conventionally synthesized magnetic nanocatalysts. These results demonstrate that the valorization route preserves the catalytic functionality of the magnetic phase while simultaneously transforming the recovered plastic into a functional component of the remediation process.

## 3. Materials and Methods

### 3.1. Chemicals and Reagents

Diethylene glycol (DEG, 99%), N-Methyldiethanolamine (NMDEA, 99%), Iron (III) chloride hexahydrate (FeCl_3_∙6H_2_O, ≥98%), iron (II) chloride (FeCl_2_ ≥ 98%), sodium hydroxide (NaOH, ≥98%), iron (III) nitrate nonahydrate (Fe (NO_3_)_3_.9H_2_O), nitric acid (HNO_3,_ 65%), sucrose (≥99.5%), Poly (ethylene terephthalate) (PET, granular), sodium dodecyl sulfate (SDS, 99%), hydrogen peroxide (H_2_O_2_, 30%), methylene blue (MB, 82%), terephthalic acid (TPA, 98%), deuterium (D_2_O, 99.9 atom % D), and 5,5-dimethyl-1-pyrroline N-oxide (DMPO, ≥97%) spin-trapping assays, EPR-grade, were purchased from Sigma-Aldrich (St. Louis, MO, USA). Ethanol (≥99%) and ethyl acetate (≥99.8%) were purchased from Scharlau (Sentmenat, Barcelona, Spain). Iron (II) acetate, anhydrous (97%), was purchased from Strem Chemicals, Inc (Newburyport, MA, USA).

### 3.2. Synthesis of Initial Magnetic Carriers: Iron Oxide Nanoflowers

The synthesis of NFs was carried out using a previously established protocol [33]. As illustrated in Figure S1A (top) in the Supplementary Materials, NFs were synthesized using a 50:50 (*w*/*w*) DEG/NMDEA solvent blend. Initially, 0.1 g NaOH was added to 6.4 g of the blend and ultrasonicated (7 min, 70 °C). Concurrently, 6.4 × 10^−4^ mol FeCl_3_·6H_2_O and 3.1 × 10^−4^ mol FeCl_2_ were dissolved in 12.8 g of the solvent mixture under magnetic stirring for 45 min. Both mixtures were then combined in a G30 microwave vial, placed inside a Monowave 300^®^ (Anton Paar GmbH, Graz, Austria) and heated to 220 °C at 0.2 °C s^−1^ (20 min hold) for nucleation and growth, followed by heating to 250 °C at 0.1 °C s^−1^ (1 h hold) for core sintering. The resulting precipitates were magnetically recovered and washed five times with a 50:50 (*v*/*v*) ethanol/ethyl acetate mixture. To ensure colloidal stability, samples underwent acidic and oxidative treatments [65]. Briefly, precipitates were washed with 10 mL of HNO_3_, then redispersed in a solution of 3 g Fe (NO_3_)_3_·9H_2_O in 6 mL of distilled water and stirred at 90 °C for 30 min. After cooling to room temperature (RT) and a second HNO_3_ wash, the final product was washed five times with ethanol/ethyl acetate and dispersed in 10 mL of distilled water.

### 3.3. Microplastics Preparation and Magnetic Harvesting

MicroPET was prepared via cryogenic treatment of commercial granular PET using liquid nitrogen (Figure S1A, bottom), followed by a 3 min grinding process using a high-speed rotatory mill. The resulting particles were manually sieved through a 300 μm mesh (W.S. Tyler) to ensure uniform particle size. Finally, the fraction was collected and stored in a sealed container for subsequent analysis.

Following an established methodology [15], the harvesting process involved the interaction between the synthesized NFs and MicroPET (mass ratio of 1) by ultrasonic mixing at pH 7 and 30 min, as depicted in Figure S1A. On the other hand, for the MIONPs synthesized using MicroPET-derived EG, the particles were mixed again with MicroPET at specific mass ratios (1, 5 and 10) at different pHs (3, 7 and 11) and then subjected to ultrasonication for different times (5,15 and 30 min) to facilitate the formation of magnetic hybrids for their later use as catalytic agents. Specific parameters were isolated for each study: pH effects were evaluated at a constant mass ratio and sonication time; mass ratio effects were tested at a fixed pH and sonication time; and sonication time effects were investigated while maintaining the pH and mass ratio at constant levels.

After treatment, magnetic hybrids were separated using a NdFeB magnet (60 × 30 mm, with a surface gradient of 320 kA m^−1^). The supernatant was filtered (0.22 µm Millipore membrane) and dried at 50 °C to quantify the MicroPET non-harvested fraction through weight difference.

### 3.4. Reusability of NFs and SC as MicroPET Carriers

A 65% (*w*/*w*) sucrose solution was used as a high-density barrier to enable separation based on density differences [35,66]. First, 100 mg of the hybrid material (mass ratio 1:1) was dispersed in 11 mL of distilled water with 1.1 mL of absolute ethanol (Figure S1B, top). The mixture was sonicated for 10 min and then transferred to a 50 mL vial. Next, 15 mL of the sucrose solution was carefully injected at the bottom of the vial, creating a sharp interface with the suspension (Figure S1B, top).

The system was centrifuged at 4500× *g* for 30 min using a soft deceleration profile. This produced distinct layers: a top aqueous phase, a concentrated band of MicroPET at the density interface, a clear sucrose layer below, and a pellet of iron-containing NFs or SC at the bottom (Figure S1B, top).

The MicroPET part was then collected with approximately 25 mL of the sucrose layer and transferred to a new vessel. This fraction was diluted with 25 mL of 0.5% SDS and warm distilled water to a final volume of 100 mL (final SDS concentration: 0.25%). The mixture was heated at 60 °C for 15 min under moderate stirring to promote detachment of residual NFs (Figure S1B, bottom).

Magnetic separation was performed by placing a magnet against the vessel wall to remove detached iron oxide nanoparticles. The resulting PET-containing supernatant was decanted and filtered under vacuum using a 0.2 µm membrane. The collected material was washed sequentially with 100 mL of distilled water (1:1 mixture at 50 °C and room temperature) and 20 mL of absolute ethanol to remove residual sucrose and surfactant, ensuring quantitative recovery of dry MicroPET (Figure S1B, bottom).

Recovered NFs or SC were washed twice with distilled water and ethanol, then dried in an oven. Finally, reusability was assessed over five consecutive cycles by reintroducing the recovered NFs or SC into subsequent separations.

### 3.5. PET Upcycling: Neutral Hydrolysis and Reagent Recovery

The upcycling of recovered MicroPET into its primary chemical reagents, TPA and EG, was conducted via neutral hydrolysis. In a typical preliminary procedure, 50 mg of MicroPET was reacted with 50 mL of distilled water in a 100 mL polyphenylene (PPL)-lined stainless-steel autoclave at 250 °C for 12 h. Following the reaction, the 50 mL crude mixture was filtered using a 0.22 μm Millipore membrane to remove the unreacted solid fraction. This retained fraction was dried at 50 °C and weighed to determine the gravimetric hydrolysis yield, and the resulting supernatant was stored for subsequent experiments. To optimize the process, the amount of MicroPET was increased to 30 g and treated in a 1 L PTFE-lined autoclave with 500 mL of distilled water at 250 °C for 24 h. Following the reaction, the crude mixture was filtered using a 0.22 μm Millipore membrane to remove the unreacted solid fraction. The obtained supernatant was acidified with HCl and filtered again through a 0.22 μm Millipore membrane to separate the precipitated TPA. The remaining filtrate was transferred to a rotary evaporator to remove excess water under controlled temperature and reduced pressure conditions. After evaporation, a 2.5 mL residue was collected and stored for analysis.

### 3.6. Synthesis of Magnetic Iron Oxide Nanoparticles with Upcycled MicroPET

The synthesis protocol was adapted from a previously reported method for NFs [33], with the primary modification being the substitution of conventional amine and polyol sources with the recovered PET hydrolysis supernatant. The mixture was subjected to microwave-assisted synthesis under the thermal parameters optimized for standard NFs (ramp rate of 0.2 °C s^−1^, reaction time and target temperature of 220 °C), omitting the second step at 250 °C. To ensure optimal colloidal stability for downstream applications, the obtained nanoparticles underwent the post-synthesis acid treatment described previously.

For comparative purposes, an alternative formulation designated as MC was also synthesized using modified reagent scales and distinct thermal parameters; the complete experimental procedure for MC is detailed in the Supplementary Materials (Section S1).

### 3.7. Degradation Experiments

MB decolorization due to degradation (100 ppm) was assessed using SC, MC and the corresponding MicroPET/ SC and MicroPET/MC hybrids (0.1–1 mg mL^−1^) under three distinct activation modes: 25 °C, 90 °C, and magnetic induction (AMF: 200 kHz, 24 kA m^−1^). After a 2 h equilibration phase, the reaction was initiated by adding H_2_O_2_ (1% (*v*/*v*) in the final volume). Conventional heating experiments utilized a thermomixer from Eppendorf^®^ (Hamburg, Germany), whereas magnetic induction was applied under mechanical agitation for 2 h using a Fives Celes AC field generator (model N° 12118 M01, Lautenbach, France). Final MB levels were determined calorimetrically using a UV-Vis Spectrophotometer WPA Biowave II+ from Biochrom (Cambridge, UK), with quantification based on a 0–5 ppm external calibration curve at λ = 660 nm.

### 3.8. Characterization Methods

#### 3.8.1. Morphological and Structural Characterization

The morphology and nanostructure of the MIONPs and the MicroPET were investigated via TEM, using a JEOL-JEM 1010 microscope (JEOL Ltd., Tokyo, Japan) operating at 100 keV and equipped with a Gatan Orius 200 SC digital camera (Gatan Inc., Pleasanton, CA, USA). Samples were prepared by drop-casting a diluted aliquot onto a copper grid. Micrographs were processed using ImageJ software (version 1.54g, National Institutes of Health, Bethesda, MD, USA), with a minimum of 200 particles measured to determine the mean size and distribution through a lognormal function fit. HRTEM was performed using a CM200 microscope (Philips Electron Optics, Eindhoven, The Netherlands) at 200 kV. Representative micrographs were processed using ImageJ software.

Further morphological details were obtained using a FEI Veiros 460 SEM, providing a spatial resolution of 0.6 nm at 2 kV and 0.7 nm at 1 kV. For SEM analysis, MicroPET powder was immobilized on a carbon adhesive tape. Elemental composition was determined via EDX using an EDAX Octane Plus detector (EDAX LLC, Mahwah, NJ, USA) integrated into the SEM instrument.

The crystal structure of the nanoparticles was identified using a Bruker D8 Advance diffractometer (Karlsruhe, Germany) with Cu Kα radiation, scanning between 2θ values of 10 and 70° at 1 °C min^−1^. The crystal size was calculated by measuring the width of the highest intensity peaks using the Scherrer equation, D = K∗λ/β∗cos θ, which was adjusted in the EVA software (version 8.0, Bruker AXS GmbH, Karlsruhe, Germany). In this equation, D represents the crystallite size, K is a constant, λ is the X-ray wavelength, β corresponds to the full width at half maximum (FWHM) of the diffraction peak corrected for instrumental broadening, and θ is the Bragg diffraction angle. The peak width (β) was determined from the most intense reflection associated with the magnetite/maghemite (311) crystallographic plane for all samples.

#### 3.8.2. Compositional Characterization

FTIR spectra were recorded on a Bruker Vertex 70V spectrometer (Bruker Optics GmbH, Ettlingen, Germany) in the 250 to 4000 cm^−1^ range. Samples were prepared as KBr pellets containing 2% (*w*/*w*) of the analyte. Raman spectra were acquired in the near infrared to reduce the fluorescence background. The laser excitation wavelength was 784.6 nm. The light was focused on the sample with a 100× microscope objective. The scattered light was collected with the same objective in a backscattering geometry. The laser power on the sample was a few milliwatts. The inelastically scattered light was selected with edge filters and analyzed with a 300 mm focal length spectrometer and a cooled CCD detector. Regarding sample preparation, the materials were analyzed directly as dry powders and liquid without any prior chemical treatment, modification, or dilution. A small amount of each sample was placed onto a silicon wafer and quartz cuvette. Finally, the laser beam was focused directly onto the surface through the microscope objective.

The TGA analysis of the nanoparticles was performed using 5 mg of sample in an SDT Q600 differential scanning calorimeter (TA Instruments, New Castle, DE, USA), heating in air at a rate of 10 °C min^−1^ until reaching a temperature of 900 °C. This process determines the number of organic compounds on the surface of the nanoparticle, specifically related to the presence of EG.

Iron content within the NF suspensions was quantified using Inductively Coupled Plasma Optical Emission Spectrometry (ICP-OES) (Optima 2100 DV, Perking Elmer, Waltham, MA, USA). The procedure involved digesting 25 µL of NF suspensions in 1 mL of aqua regia at 90 °C overnight, followed by dilution to a final volume of 25 mL with distilled water.

The chemical identity of the recovered liquid phase and identification of co-existing species were studied through ^1^H-NMR spectroscopy. Samples (8 mg) were dissolved in 0.7 mL of D_2_O, transferred to a 5 mm NMR tube, and analyzed on a Bruker AVIII HD 300 MHz spectrometer (Bruker BioSpin GmbH, Ettlingen, Germany) with a BACS-60 autosampler at 298 K. A total of 256 scans were acquired. Chemical shifts are reported in ppm relative to the residual solvent peak (7.26 ppm).

#### 3.8.3. Colloidal and Colligative Properties

The colloidal characterization of the nanoparticles was performed using a ZetaSizer Ultra system (Malvern Panalytical, Malvern, UK) equipped with a 633 nm He-Ne laser (maximum power of 4 mW) and a fixed measurement angle of 173° with the nanoparticles previously diluted and sonicated. The surface charge behavior of the nanoparticles was evaluated by measuring the ζ-potential across a wide pH range. The pH was adjusted with KOH or HNO_3_ solutions, while a 10^−3^ M KNO_3_ solution was used as the background electrolyte to ensure a constant ionic strength throughout the measurements.

The osmolality of the EG-rich supernatant was determined by freezing-point depression osmometry using a Micro-Osmometer Model 3320 (Advanced Instruments, Norwood, MA, USA). All measurements were performed using 20 μL sample volumes, with the system calibrated using NIST-traceable standards (50 and 850 mOsm kg^−1^ H_2_O).

#### 3.8.4. Magnetic Properties

Steady-state magnetic characterization was performed using a Quantum Design MPMS-3 SQUID magnetometer (Quantum Design Inc., San Diego, CA, USA), with hysteresis loops recorded at 5 K and 290 K under a maximum applied field of 4000 kA m^−1^. Furthermore, the sample mass was adjusted based on the percentage of organic matter determined via TG analysis. Finally, saturation magnetization (M_s_ = M at maximum field) and coercivity (H_c_) were derived from these hysteresis curves using Origin 2018 software.

The heating efficiency of the SC, MC NFs and the corresponding MicroPET/NF hybrids was evaluated by recording the temperature–time profiles under an AMF using a FIVES CELES induction system and different frequencies and fields: frequencies of 96 and 200 kHz with magnetic fields from 8 to 48 kA m^−1^. Aqueous dispersions (1 mg mL^−1^) were monitored using an FTX fiber optic temperature sensor from Osensa Innovations (Burnaby, BC, Canada). The SAR was calculated from the initial slope of the heating curve as SAR = (C_p_/c) (dT/dt), where C_p_ is the heat capacity of the medium (4185 J L^−1^ K^−1^), c is the nanoparticle concentration (g L^−1^), and dT/dt is the initial temperature increase rate (°C s^−1^).

Magnetophoretic behavior was assessed using a SEPMAG LAB M system (Sepmag Systems S.L., Cerdanyola del Vallès, Barcelona, Spain), which monitors nanoparticle migration via turbidity detection. Prior to analysis, suspensions (5.5 mg NPs mL^−1^) were sonicated for 30 min. Separation was conducted under a magnetic induction gradient of ∇B = 24 T m^−1^ in vials with a radius L = 0.007 m (corresponding to a magnetic field H_max_ = 1.337 × 10^5^ A m^−1^). The suspended fraction was calculated as fₙ = (P_n_ − H_m_)/ (P_i_ − H_m_), where P_n_ is the homogeneity at time n, H_m_ is the minimum homogeneity after complete separation, and P_i_ is the initial homogeneity value. Assuming magnetic saturation, the kinetics follow f_n_ 1/2 = 1 − (v_s_/L)t (with t being the analysis time). The terminal velocity (v_s_) was obtained from the slope of √fₙ versus time. Finally, the magnetophoretic mobility (Uₘ) was determined as U_m =_ v_s_/(H_max_·dB/dr), using the separator constant H_max_·dB/dr = 3.21 × 10^6^ A·T m^−2^ with results expressed in m^3^ T^−1^ A^−1^ s^−1^.

#### 3.8.5. Identification of Free Radical Production by EPR

The generation of free radicals by the SC sample was studied by EPR using BRUKER ELEXSYS II-E500 spectrometer (Bruker BioSpin GmbH, Karlsruhe, Germany) working in the X-band (9.5 GHz) at room temperature, using the nitrone-based spin trap DMPO. The nanoparticles were dispersed in distilled water at a concentration of 1 mg/mL, and the suspension was adjusted to pH 5 by adding drops of 0.1 M HCl. An aliquot of 100 µL of this suspension was mixed with 25 µL of a DMPO solution in water (1 g: 6 mL) and 5 µL of 30% H_2_O_2_ aqueous solution. The mixture was immediately transferred to a quartz capillary and measured by EPR. A control experiment (blank) was performed following the same protocol in the absence of nanoparticles. The reaction time was counted from the addition of H_2_O_2_, and spectra were acquired at intervals of no more than 10 min over a total reaction time of 1 h.

The identification of the free radicals generated during the reaction was performed by fitting the EPR spectra using the NIEHS Spin-Trap database [57] for the spin-trapping species. The area of the EPR resonance signal (double integral of the EPR spectrum) is proportional to the concentration of the corresponding paramagnetic species. Therefore, the relative radical concentrations were determined by comparing the integrated area for each species, obtained from the fitted EPR spectra, with that of a pattern sample (MgO: Mn^2+^ crystal). The fitting was performed using the SpinFit module of the Xepr software (version 2.6b.146; Bruker BioSpin GmbH, Karlsruhe, Germany) and the hyperfine coupling constants, a_N_ and a_H_, were reported for the corresponding DMPO adduct. The hyperfine coupling constants, a_N_ and a_H,_ describe the interaction between the unpaired electron of the DMPO adduct and the magnetic nuclei of the neighboring atoms of nitrogen (I_N_ = 1) and hydrogen (I_H_ = 12). For the DMPO-N adduct, the hyperfine parameters were a_N_ = 14.9 G; for DMPO-OH, a_N_ = 14.8 G and a_H_ = 14.6 G; and for DMPO-OOH, a_N_ = 14.05 G, a_H(β)_ = 11.12 G and a_H(γ)_ = 1.27 G. From these fits, the relative concentration of each species was obtained as a function of reaction time.

## 4. Conclusions

This work demonstrates a circular approach for the remediation and valorization of MicroPET, in which plastic contaminants are not only removed from water but transformed into functional materials for subsequent environmental applications. By combining magnetic harvesting, neutral hydrolysis, and nanoparticle synthesis, we established a route connecting PET waste recovery with the production of new magnetic nanomaterials.

A key outcome of this study is the demonstration that PET hydrolysis products can be successfully reintegrated into the synthesis of magnetic nanoparticles, yielding recycled materials that retain both harvesting and catalytic functionalities. The resulting secondary clusters efficiently captured MicroPET and acted as AMF-responsive heterogeneous Fenton catalysts, illustrating how plastic-derived feedstocks can be converted into active materials for water remediation.

Beyond demonstrating individual processing steps, the present work establishes the feasibility of linking plastic recovery, chemical depolymerization, and material re-engineering within a single framework. Although high PET conversion was achieved during hydrolysis, monomer recovery during scale-up remained limited by reactor configuration and downstream separation losses, highlighting the need for improved mixing and product recovery strategies. In addition, the circular concept was demonstrated through a single recycling cycle under controlled laboratory conditions, and further studies will be required to evaluate repeated cycles, more complex plastic waste streams, and real wastewater matrices.

Nevertheless, the successful integration of MicroPET capture, PET depolymerization, and nanoparticle re-synthesis demonstrates that plastic waste can be transformed from an environmental contaminant into a valuable resource for advanced remediation technologies, providing a promising foundation for future circular water-treatment strategies.

## Figures and Tables

**Table 1:** Comparison between unscaled and scaled MicroPET hydrolysis experiments.

	PET		TPA		EG	
Experiment	Intial Mass (g)	Conversion (%)	Theoretical (g)	Yield (g, %)	Theoretical (g)	Yield (g, %)
Unscaled hydrolysis *	0.05	97	0.043	N/D ^§^	0.016	N/D ^§^
Scaled hydrolysis ^†^	30	28.7 ^‡^	25.93	7.44 (28.7)	9.69	2.78 (28.7)

* Reactor volume: 100 mL/ water volume: 50 mL; ^†^ Reactor volume: 1000 mL/ water volume: 500 mL; ^‡^ Estimated from the recovery yield of TPA and EG; ^§^ N/D = not determined.

**Table 2:** Recent developments in removing MicroPET using iron-based magnetic carriers.

Magnetic Carrier	Particle Size (nm)	MicroPET Size (μm)	Harvesting Ratio (mg g^−1^)	Reference
Fe_3_O_4_ disks	≈450	10	188.4	[46]
Fe_3_O_4_MPs*@HDTMS	≈120,000	38–75 and 75–150	1000 **	[47]
Pristine Fe_3_O_4_	≈10	≈80	67 *	[48]
Fe_3_O_4_NPs @HDTMS	25	1000–8000	1000 *	[49]
Nano-Fe@ZIF-8	<100 nm	≈200	25–165 *	[50]
γ-Fe_2_O_3_ NFs, SC	39 and 29	370	1000	This work

* MP = microparticles; ** Estimates based on the weight and length data provided for the MicroPET.

**Table 3:** Magnetic parameters of NFs, SC and the corresponding MicroPET/SC composite.

Sample	M_S_ (Am^2^ kg^−1^)		H_c_ (kA m^−1^)		M_r_/M_S_		SAR (W g _Fe_^−1^)	
	290 K	5 K	290 K	5 K	290 K	5 K	96 kHz	200 kHz
NFs	89	98	0.7	15.8	0.01	0.22	423	710
SC	57	67	3.4	12	0.07	0.20	164	230
MicroPET/SC	33	38	0.71	12	0.02	0.24	—	—

## Data Availability

Data is contained within the article and Supplementary Materials.

## Supplementary Materials

The following supporting information can be downloaded at: https://www.mdpi.com/article/10.3390/molecules31173117/s1, Figure S1: Schematic representation of experimental methodology of the synthesis of NFs and magnetic separation of MicroPET; Figure S2: Structural and morphological characterization of NFs; Figure S3: Magnetic properties and hyperthermia performance of the synthesized NFs; Figure S4: Frequency histogram illustrates the particle size distribution of MicroPET width; Figure S5: Effect of hydrolysis temperature on MicroPET depolymerization under unscaled conditions (50 mg PET, 50 mL water, 100 mL reactor); Figure S6: Comparative characterization of IR (S6A) and Raman intensity (S6B) profiles for commercial (green) and MicroPET derived TPA (orange); Figure S7: (A–E) Characterization of the MC; Figure S8: Morphological, compositional, and operational characterization of the MicroPET/MC hybrid assemblies; Figure S9: Magnetic and magneto-thermal characterization of the carriers; Figure S10: MB degradation efficiency via a heterogeneous Fenton-like reaction activated by H_2_O_2_ using pristine MC and MicroPET/MC hybrid; Figure S11: Representative EPR spectra and fit of the blank, the as-synthesized SC sample before (BAT) and after (AAT) acidic treatment; Table S1: Magnetic parameters of bare MC and the corresponding MicroPET/MC composite; Table S2: Representative examples of AMF-assisted heterogeneous Fenton and Fenton-like degradation processes reported in the literature for different classes of contaminants and environmental matrices. References [15,33,34,51,61,62,63,64,67,68,69,70,71,72,73] are cited in the supplementary materials.
