# Supplementary material for: From Microplastics to Multifunctional Magnetic Nanomaterials: A Circular Strategy for Water Remediation
Source: Molecules. 2026 Sep 5;31(17):3117. doi: 10.3390/molecules31173117 (PMC13567545; doi:10.3390/molecules31173117)
Supplement: Supplementary file 1 [file molecules-31-03117-s001.zip › molecules-4479800-supplementary.pdf]

molecules

Supporting Information

Article

From Microplastics to Multifunctional Magnetic Nanomaterials: A Circular Strategy for Water Remediation

Rafael Herrera-Aquino[1]*, Sabino Veintemillas-Verdaguer[1], Fernando Agulló Rueda[1], Fernanda Lyzeth Rivera[2], Nahuel Nuñez[3,4,5], Fernando Martín-Garrido[6], Helena Gavilan[6], Elin L. Winkler[3,4,5], María del Puerto Morales[1]*, Alvaro Gallo-Cordova[1]*

[1] Instituto de Ciencia de Materiales de Madrid, ICMM/CSIC, C/Sor Juana Inés de la Cruz 3, 28049 Madrid, Spain. sabino@icmm.csic.es (S.V.V), f.agullo.rueda@csic.es (F.A.R).
[2] FIM, Universidad Autónoma de Baja California, av. Alvaro Obregón s/n, Nueva, 21100 Mexicali, B.C., México.fernanda.rivera.saldivar@uabc.edu.mx (F.R.S).
[3] Departamento Magnetismo y Materiales Magnéticos, Gerencia de Física, Centro Atómico Bariloche, 8400 San Carlos de Bariloche, Río Negro, Argentina. nahuel.nunez@ib.edu.ar
[4] Instituto de Nanociencia y Nanotecnología (CNEA CONICET), Nodo Bariloche, 8400 San Carlos de Bariloche, Río Negro, Argentina. elin.winkler@ib.edu.ar (E.L.W.).
[5] Instituto Balseiro, CNEA-UNCuyo, Av. Bustillo 9500 San Carlos de Bariloche, Río Negro, Argentina.
[6] Departamento de Química Física, Facultad de Ciencias Químicas, Universidad Complutense de Madrid (UCM), Ciudad Universitaria s/n, 28040 Madrid, Spain. femart10@ucm.es, hgavilan@ucm.es

* Correspondence: rafael.herrera@icmm.csic.es (R.H.A); puerto@icmm.csic.es (M.P.M), alvaro.gallo@csic.es (A.G.C.) Tel.: +34 917166058. Ext.437266

Experimental methodology of the synthesis of NFs and magnetic separation of MicroPET

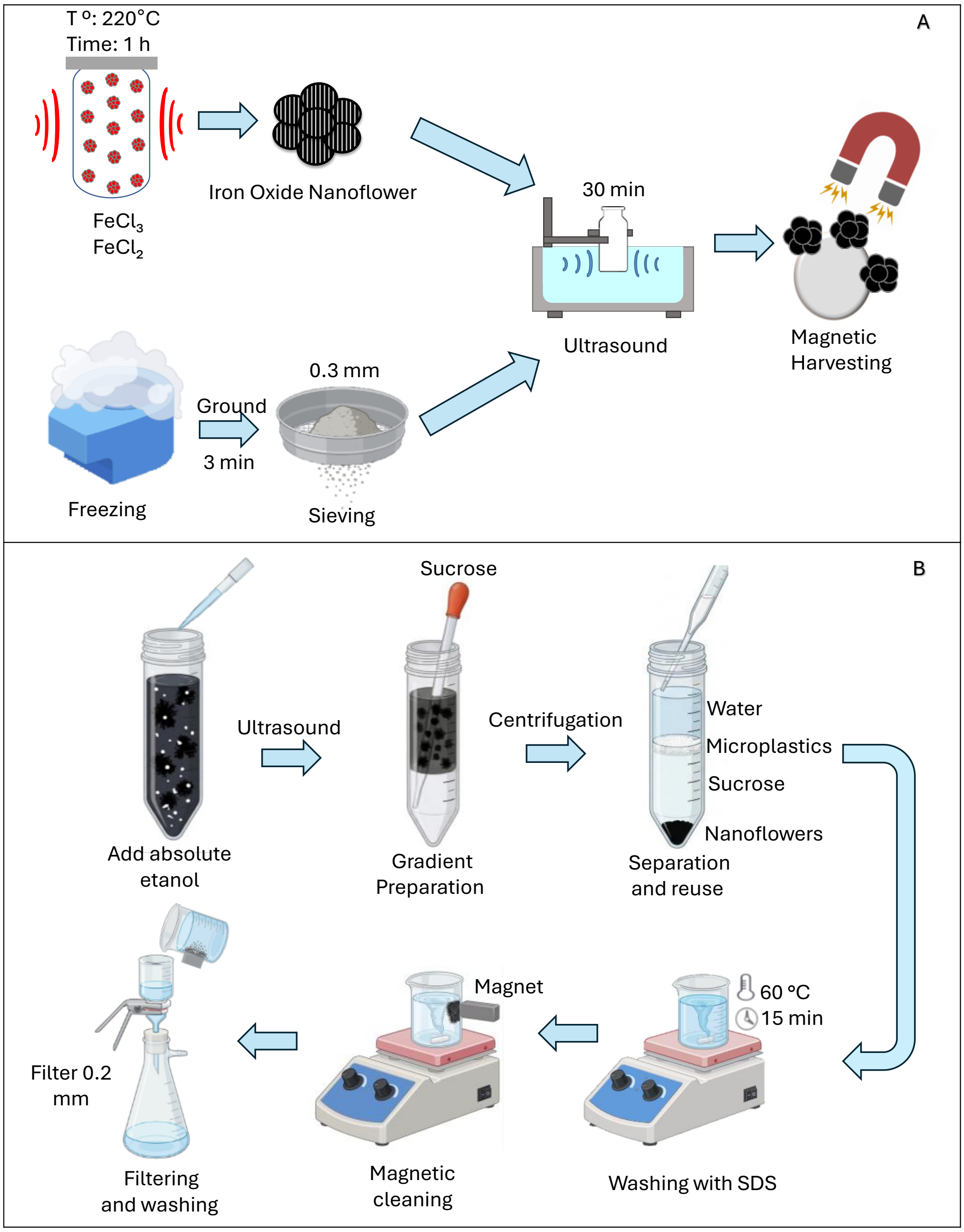

Figure S1. Schematic representation of experimental methodology of the synthesis of NFs and magnetic separation of MicroPET. (A) Synthesis of NFs from iron precursors ($FeCl_3$ and $FeCl_2$) via thermal treatment (220 °C, 1 h), followed by the ultrasound-assisted capture and magnetic harvesting of previously cryo-milled and sieved MicroPET. (B) Separation protocol for the hybrid system and nanoparticle recovery; the process involves dispersion in absolute ethanol, density gradient separation with sucrose via centrifugation, and washing cycles with SDS along with magnetic cleaning for the reuse of the NFs.

Section S1. Supporting Methodology

Synthesis of multicore-like MIONPs

The synthesis of multicore-like (MC) MIONPs was performed by adapting a previously reported protocol [1], with specific modifications to the reagent scales. Briefly, 31.30 mg of Iron (II) acetate was dissolved in a mixture of 1.91 mL of PET-derived supernatant and 0.07 mL of deionized water, resulting in a total reaction volume of 2.5 mL. The solution was transferred to a glass vial and placed in a microwave reactor. The reactor was heated at a constant rate of 3.75 °C/min until reaching 170 °C, where it was maintained for 2 h, followed by rapid cooling to room temperature. The resulting product was washed five times with a 1:1 (v/v) ethyl acetate/ethanol mixture and redispersed in distilled water via ultrasonication. Following the reaction, the nanoparticles underwent the identical post-synthesis acid treatment described in the main text to ensure optimal colloidal stability.

Section S2: NFs characterization

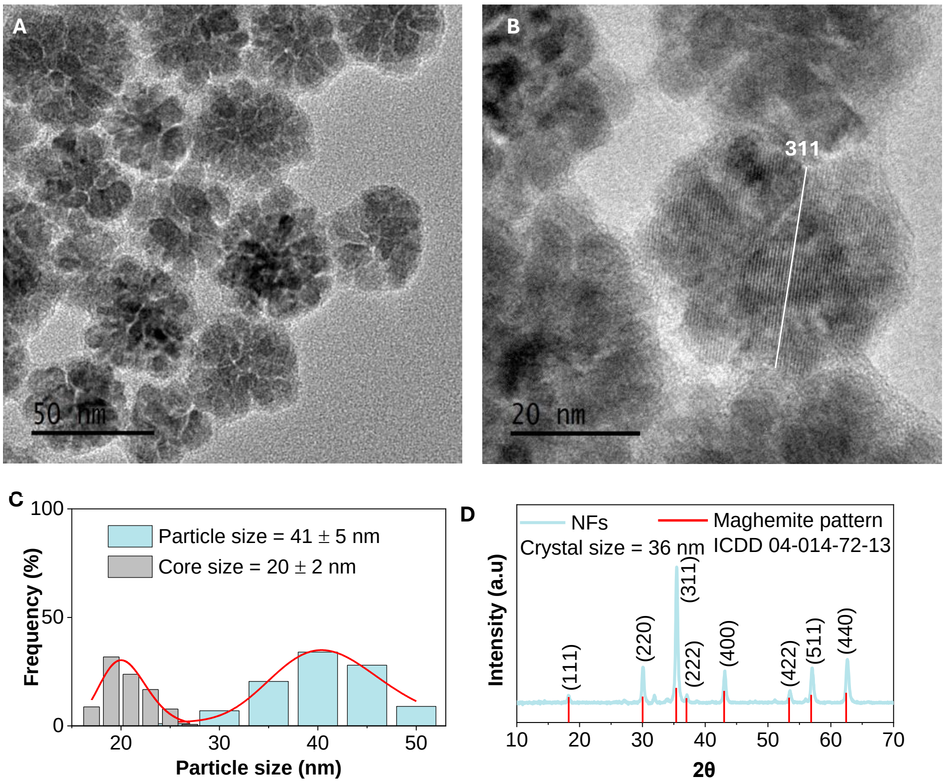

Figure S2. Structural and morphological characterization of NFs. (A) HR-TEM image showing the characteristic flower-like morphology. (B) HRTEM image at higher magnification revealing continuous lattice fringes corresponding to the (311) plane, indicative of mesocrystal formation. (C) Size distribution histograms for the external particle diameter (41 ± 5 nm) and primary core size (20 ± 2 nm). (D) XRD pattern of the NFs compared to the standard maghemite reference (ICDD 04-014-72-13).

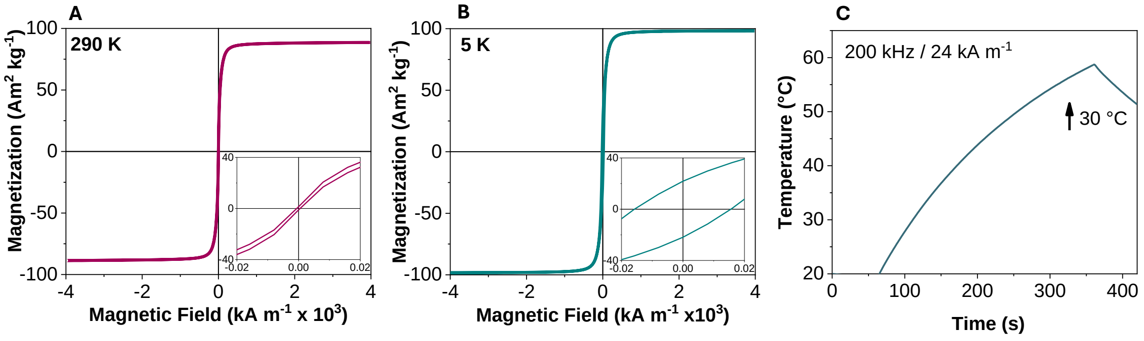

Figure S3. Magnetic properties and hyperthermia performance of the synthesized NFs. Magnetic hysteresis loops measured at (A) 290 K and (B) 5 K. The insets show an enlarged view of the low-field region, highlighting the change of regime from a superparamagnetic-like behavior at room temperature to a highly coercivity at low temperature. (C) Calorimetric heating curve demonstrating the temperature evolution of the NFs over time under an AMF (200 kHz, 24 kA/m).

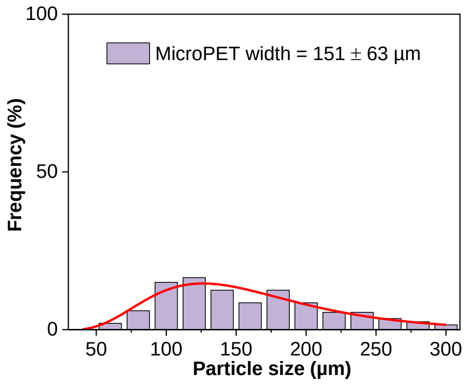

Figure S4. Frequency histogram illustrates the particle size distribution of MicroPET width. The relative frequency (%) is presented on the y-axis, and the particle size in micrometers (µm) on the x-axis. A text label in the figure inset indicates a calculated mean MicroPET width of 151 ± 63 µm, and a red solid line represents a statistical fit to the distribution data.

Section S3. Hydrolysis of PET (Influence of temperature)

To evaluate the influence of temperature on PET depolymerization and identify the optimal hydrolysis conditions, preliminary unscaled experiments were performed at different temperatures. The resulting PET conversion was quantified gravimetrically and used to select the most suitable temperature for the subsequent scaled hydrolysis experiments aimed at product recovery and valorization.

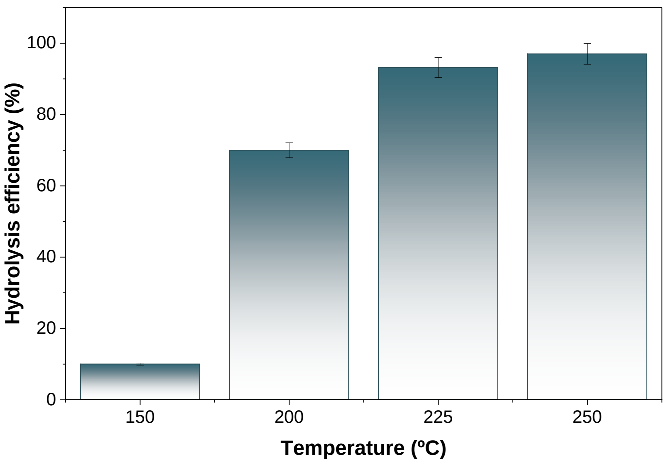

Figure S5. Effect of hydrolysis temperature on MicroPET depolymerization under unscaled conditions (50 mg PET, 50 mL water, 100 mL reactor). PET conversion was determined gravimetrically after hydrolysis at 150, 200, 225, and 250 °C. Conversion increased progressively with temperature, reaching a maximum value of 97% at 250 °C. Error bars represent the standard deviation of three independent experiments.

Section S4. Characterization of MicroPET derived TPA

Figure S6 presents the comparative vibrational characterization of the synthesized and commercial TPA. Figure S6A details the FTIR spectra, where the experimental TPA (orange) is evaluated against a high-purity benchmark (green). The experimental sample exhibits the characteristic bands of an aromatic dicarboxylic acid, notably a broad absorption region between 3521 and 3304 cm^{-1} associated with O-H stretching vibrations, and a sharp carbonyl (C=O) stretching peak at 1558 cm^{-1}[2,3]. Additional diagnostic bands include the O-H bending and C-O stretching modes from 1441 to 1396 cm^{-1}. Moving into the fingerprint region, the zone spanning 1300 to 1017 cm^{-1} encompasses multiple coupled vibrational modes; the higher frequency signals near 1285–1300 cm^{-1} are primarily attributed to the C–O stretching of the carboxylic acid group coupled with in-plane O–H deformations, whereas the bands descending toward 1017 cm^{-1} correspond to the in-plane C–H bending deformations of the aromatic ring [4]. Furthermore, the prominent signal at 740 cm^{-1} represents the out-of-plane C–H bending, which serves as a structural hallmark for the para-disubstituted benzene ring [3]. Finally, the low-frequency band detected at 518 cm^{-1} is ascribed to the out-of-plane rocking deformations of the carboxyl group (O–C=O), which typically manifest coupled with the skeletal deformations of the aromatic ring [4]. In contrast, Figure S6B provides the Raman spectra, which acts as a complementary structural probe. This highlights high-intensity modes at 1638 cm^{-1} related to the aromatic skeleton and C=O vibrations, in plane ring deformations between 1188 and 1126 cm^{-1}, and a distinctive ring-breathing mode at 840 cm^{-1}.[5,6]. Finally, the sharp peak at 264 cm^{-1} is assigned to the in-plane bending deformation of the central aromatic ring attached to the carboxylic acid group [4]. The integration of both sections revels a significant broadening and shifting of the hydroxyl bands in Figure S6A for the experimental sample, while the signals in Figure S6B remain virtually identical to the commercial standard. This divergence arises because Figure S6A is highly sensitive to changes in the molecular dipole moment, marking it susceptible to the disordered hydrogen-bonding networks or residual moisture resulting from the PET hydrolysis and recovery process, a phenomenon widely documented in recent chemical recycling studies [7]. Conversely, Figure S6B probes changes in polarizability

focusing on the symmetric, non-polar aromatic core, which remains structurally unyielding regardless of surface hydration[8]. The structural stability observed in Figure S6B confirms the successful synthesis of the TPA molecular framework, while the deviations in Figure S6A primarily reflect the specific hydration state of the sample or crystalline morphology.

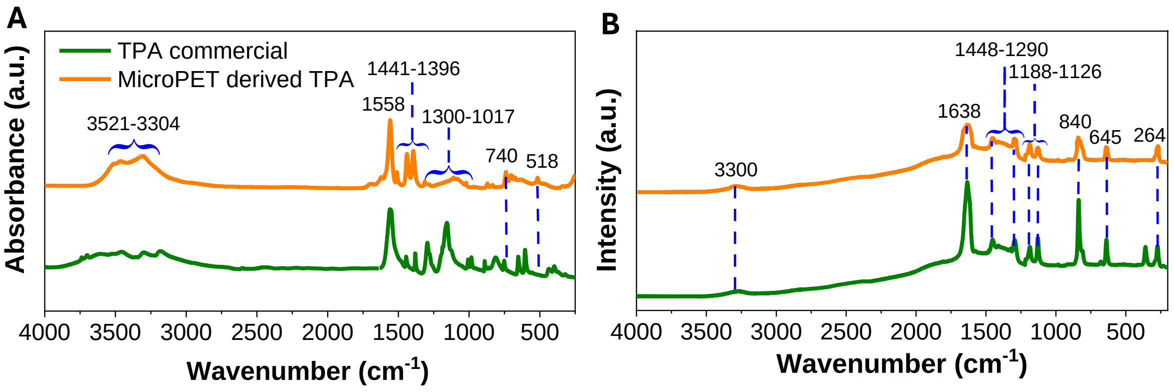

Figure S6. Comparative characterization of IR (S6A) and Raman intensity (S6B) profiles for commercial (green) and MicroPET derived TPA (orange). Key diagnostic spectral features are highlighted with guides for specific peaks, such as the broad O-H stretching band (3521-3304 cm^{-1}) and the carbonyl peak (1558 cm^{-1}) in the IR, and the aromatic ring-breathing mode (840 cm^{-1}) and the aromatic skeletal vibration (1638 cm^{-1}) in the Raman.

Section S5. Characterization of MC

Beyond the primary MIONPs described in the main text, the synthesis protocol also promoted the formation of MC. TEM analysis (Figure S7A) shows that these structures consist of multiple maghemite cores (10 ± 5 nm) assembled into larger secondary aggregates with an average size of 60 ± 14 nm (Figure S7B). Despite this higher degree of aggregation and significant organic coordination, the XRD pattern (Figure S7C) confirms that the magnetic core remains structurally intact as maghemite.

The FTIR spectrum (Figure S7D) displays Fe–O lattice bands shifted to a broader range (456–746 cm^{-1}), a sharp peak at 1017 cm^{-1} corresponding to C–O–C stretching from trapped EG, and the characteristic broad O–H stretching band at 3420 cm^{-1} [1]. These findings demonstrate that the recovered supernatant actively modulates the assembly into aggregate forms. Additionally, the TGA analysis of these aggregates (Figure S7E) shows a significantly higher organic content than the SC counterparts, with a total weight loss exceeding 39% across three distinct thermal degradation stages. This high organic content is strongly corroborated by the FTIR spectrum of the MC sample, which exhibits intense, broadened bands between 1385 and 1570 cm^{-1} (Figure S7D). These signals are derived from the supernatant and are attributed to residual TPA that was not fully removed during purification.

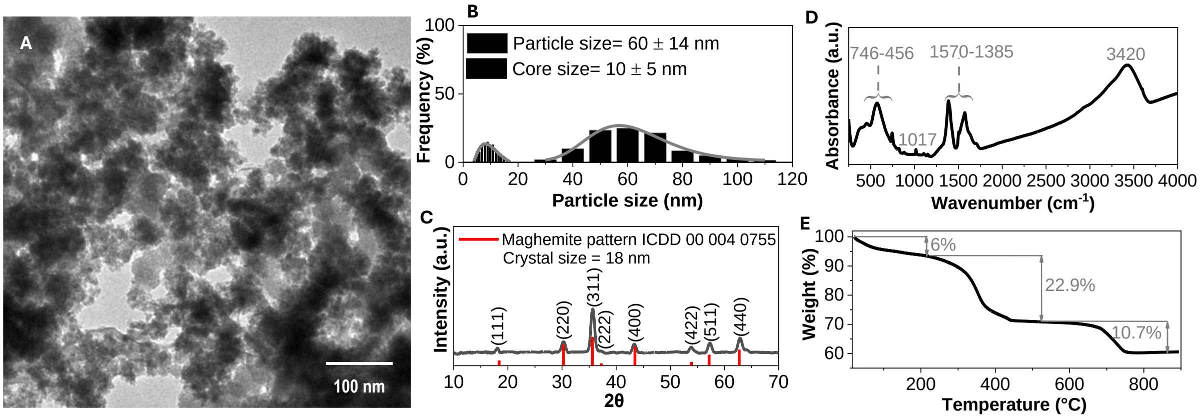

Figure S7. (A-E) Characterization of the MC: (A) TEM micrograph revealing cluster and the total aggregate, (B) particle size distribution, (C) XRD pattern validating the retention of the magnetic crystalline phase. (D) FTIR spectrum, (D) displaying the functional groups of the stabilizing coating and (E) TGA curve showing a higher organic content.

Section S6. Hybridization of MicroPET with MC by Magnetic Harvesting

Figure S8 presents the morphological and compositional characterization of the hybrid assemblies formed by the MC and the MicroPET, providing the empirical evidence necessary to demonstrate their successful interfacial integration and validate the harvesting mechanism. Specifically, the electron microscopy micrographs (Figure S8A, TEM and S8B, SEM) illustrate the architecture of these agglomerates, revealing the surface morphology where it is clearly observed how MC are firmly attached and distributed over the irregular matrix of the MicroPET. Complementarily, the EDX analysis and its corresponding elemental mapping (Figure S8C and S8D) confirm the chemical composition of the hybrid material, evidencing the uniform distribution and co-localization of the main constitutive elements; this highlights the C signal originating from the MicroPET, along with the Fe and O typical of the iron oxide phase. Following this structural validation, the operational dynamics governing the formation of these MicroPET/MC hybrids were evaluated, revealing that the assembly process is predominantly driven by the contact duration and the polymer-to-magnetic agent mass ratio, exhibiting minimal sensitivity to the initial pH of the aqueous medium. Increasing the proportion of polymer relative to the MC continuously enhances surface coverage until a saturation point is reached, while extending the incubation period prior to magnetic extraction significantly boosts the overall stability of the composite. Notably, under optimized experimental settings, specifically a neutral pH, a 30-minute interaction phase, and an equivalent 1:1 mass ratio, a maximum harvesting ratio of 1000 mg of MicroPET per gram of MC was achieved. This outstanding extraction capacity is practically identical to the peak performance recorded for the SC system, demonstrating a highly consistent and reproducible extraction mechanism across different iron oxide platforms. At this optimized operational equilibrium, the reduction of electrostatic repulsive forces among the MC particles presumably encourages interparticle agglomeration, thereby promoting the structural expansion of the hybrid composite networks in a way that synergistically complements the fundamental interfacial binding forces driving the efficient capture of the MicroPET.

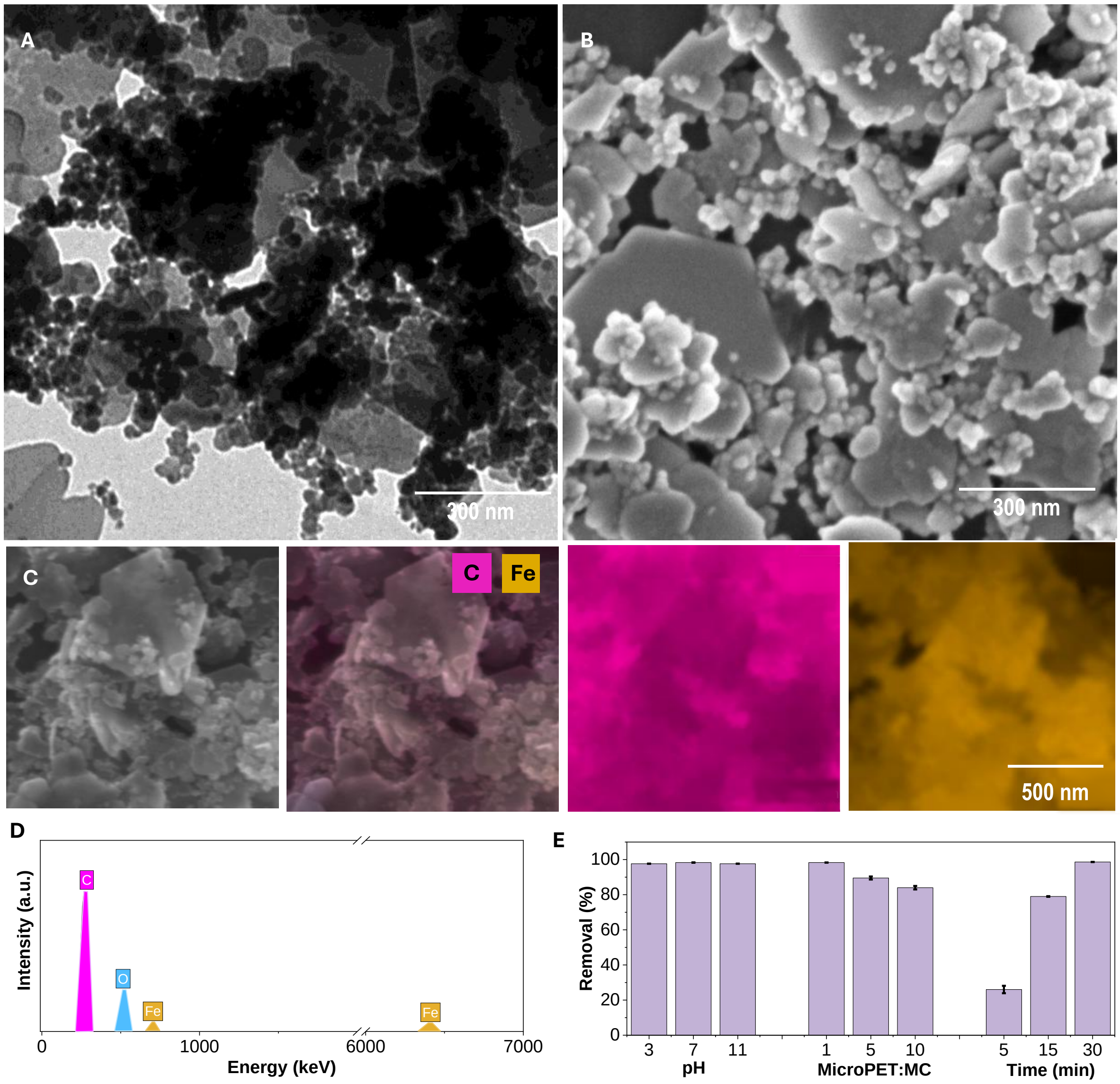

Figure S8. Morphological, compositional, and operational characterization of the MicroPET/MC hybrid assemblies. (A, B) Electron microscopy images (TEM and SEM) illustrating the surface morphology and the firm interfacial adhesion of the MC onto the irregular MicroPET matrix. (C, D) EDX spectrum and corresponding elemental mapping confirming the uniform distribution and spatial co-localization of C, Fe across the hybrid structure. (E) Operational evaluation revealed a maximum harvesting capacity of 1000 mg of MicroPET per gram of MC (under optimized conditions of neutral pH, 30-minute incubation, and a 1:1 mass ratio), matching the peak performance observed for the SC system.

Section S7. Magnetic Properties of MC and MicroPET/MC hybrid

The field-dependent magnetization curves (hysteresis loops) for the samples are illustrated in Figures S9A-B, showing the relationship between magnetization and the applied magnetic field. At 290 K, all materials exhibit a quasi-superparamagnetic behavior defined by nearly negligible H_c and M_r, with the M_s decreasing from the bare MC to the composite architecture (MC > MicroPET/MC hybrid). A detailed summary of these extracted magnetic parameters (M_s, M_r, and H_c) is compiled in Table S1. At 290 K, this superparamagnetic-like state is corroborated by low coercivity values (0.92 to 3 kA m^{-1}) and minimal remanence ratios (M_r/M_s spanning 0.01 to 0.07). Conversely, cooling the specimens to 5 K induces a pronounced magnetic blocking effect, resulting in a significant enhancement of M_s, H_c, and M_r compared to their room-temperature counterparts. This low-temperature blocking is validated by a sharp increase in H_c up to 16 kA/m and a rise in the M_r M_s^{-1} ratio to 0.20; however, this value remains below the theoretical threshold of 0.5 expected for randomly oriented, non-interacting single-domain particles [9]. Lastly, the notable reduction in M_s observed upon hybrid formation is directly attributed to the significant mass contribution of the non-magnetic organic polymeric matrix (MicroPET) covering the active magnetic surface.

The iron-normalized SAR (expressed in W g_{Fe}^{-1}) for the pristine MC is plotted against the applied magnetic field intensity in Figure S9C. Under a magnetic field strength of 24 kA m^{-1}, the MC exhibits a markedly superior magneto-thermal response at an operating frequency of 200 kHz compared to 100 kHz, reaching a value of 160 W g_{Fe}^{-1} versus 115 W g_{Fe}^{-1}, respectively. This pronounced frequency-dependent increase indicates that the 200 kHz condition successfully optimizes the dominant magnetic relaxation pathways specifically within these isolated single-core structural configurations. Interestingly, the heating capacity of 160 W g_{Fe}^{-1} achieved by the pristine MC under these optimal conditions outperforms the 60 nm multi-core aggregates (NF60) reported by [1], which were limited to a lower SAR of 144 W gFe^{-1}. This lower heating performance in their disordered clusters is typically attributed to random core assembly, where negative intra-aggregate dipolar interactions frustrate the overall magnetization of the system under an AC field.

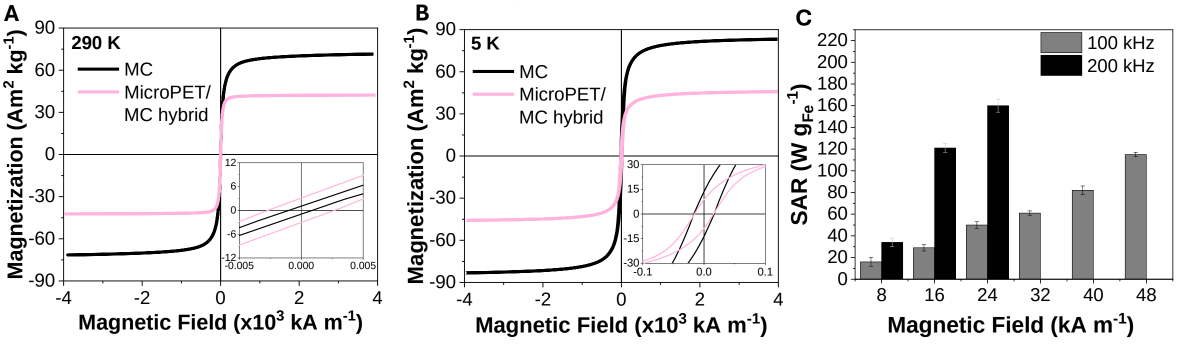

Figure S9. Magnetic and magneto-thermal characterization of the carriers. (A, B) Field-dependent magnetization curves (hysteresis loops) for bare MC and the MicroPET/MC hybrid measured at 290 K and 5 K, showing quasi-superparamagnetic behavior at 290 K and a transition to a blocked state at 5 K. (C) SAR as a function of the applied magnetic field for the pristine carrier at operating frequencies of 100 kHz and 200 kHz, illustrating the frequency-dependent optimization of magnetic relaxation pathways.

Table S1. Magnetic parameters of bare MC and the corresponding MicroPET/MC composite

Sample	Ms ($Am^2\ kg^{-1}$)		Hc ($x10^3$ kA m^{-1})		$M_r\ M_s^{-1}$		SAR ($W\ gFe^{-1}$)	
	290 K	5 K	290 K	5 K	290 K	5 K	96 kHz	200 kHz
MC	73	80	0.92	16	0.01	0.17	115	230
MicroPET/MC	43	44	3	15	0.07	0.20	—	—

Section S8. Application of MC and MicroPET/MC Hybrid

The performance of the heterogeneous fenton-like catalytic system activated by H_2O_2 for MB removal is presented in Figure S10. At a lower catalyst dosage of 0.1 mg mL^{-1}, the degradation remains negligible (<10%) regardless of the external stimulus; however, elevating the catalyst concentration to 1.0 mg mL^{-1} markedly enhances the reaction kinetics, highlighting the necessity of achieving a threshold active-site density for efficient reactive reactive oxygen generation. At 25 °C, both the pristine MC and the MicroPET/MC hybrid exhibit modest catalytic activities, yielding degradation efficiencies between approximately 10% and 37%. In contrast, conventional thermal activation at 90 °C significantly drives the reaction forward, achieving efficiencies of ~50–70% as the thermal input successfully surmounts the activation energy barrier of the Fenton process. When subjected to an alternating magnetic field (AMF: 200 kHz, 24 kA m^{-1}), the system achieves a degradation range of ~25–43%, with the MicroPET/MC hybrid demonstrating a notable ~22% improvement in catalytic performance compared to its bare counterpart (90°C). As detailed in the main manuscript, this catalytic enhancement is driven by localized magneto-thermal phenomena and surface pre-concentration of MB on the MicroPET matrix, which synergistically optimize the advanced oxidation stages at the active iron centers.

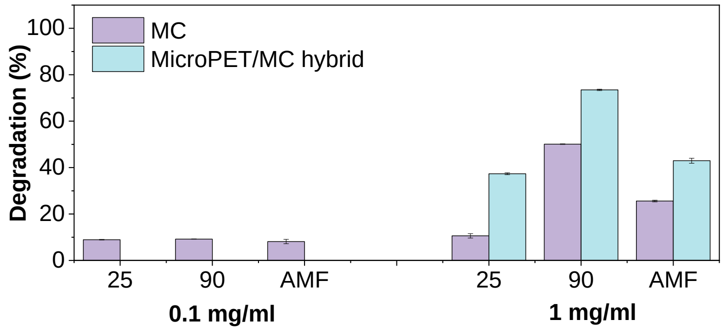

Figure S10. MB degradation efficiency via a heterogeneous fenton-like reaction activated by H_2O_2 using pristine MC and MicroPET/MC hybrid. The performance is compared under various operational stimuli, at 25 °C, conventional 90 °C, and AMF (200 kHz, 24 kA m^{-1}) at catalyst dosages of 0.1 mg/mL and 1.0 mg mL^{-1}, highlighting the enhanced performance of the hybrid assembly driven by localized surface phenomena.

Section S9. Degradation of organic compounds

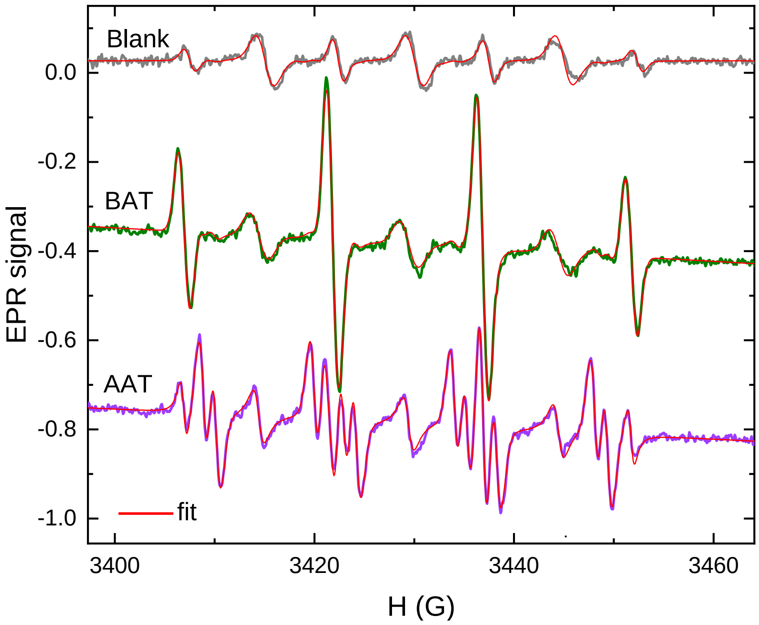

Figure S11. Representative EPR spectra and fit of the blank, the as-synthesized SC sample before (BAT) and after (AAT) acidic treatment.

Table S2. Representative examples of AMF-assisted heterogeneous Fenton and Fenton-like degradation processes reported in the literature for different classes of contaminants and environmental matrices.

Pollutant / Matrix	Magnetic-field-assisted process	AMF conditions	Reaction time (min)	Initial Pollutant concentration	Decolorization (%)	Mineralization (%)	Ref.
Methylene Blue	Fe_3O_4 nanoparticles + Electro Fenton + AMF	H = 150 G	90	20 mg L^{-1}	46	N/R*	[10]
Tetracycline	$MnFe_2O_4$ nanofibers + H_2O_2 + AMF	H = 32 kA m^{-1}; f = 450 kHz	1200	0.32 ug mL^{-1}	50	N/R*	[11]
Bisphenol A	Fe@$NiFe_2O_4$ nanoparticles + H_2O_2 + AMF	f = 222 kHz	15	20 mg L^{-1}	100	N/R*	[12]
Methylene Blue	Maghemite nanoparticles + H_2O_2 + AMF	H = 16 kA m^{-1}; f = 200 kHz	120	500 mg L^{-1}	100	N/R*	[1]
Acid Orange 8	Maghemite nanoparticles + H_2O_2 + AMF	H = 16 kA m^{-1}; f = 200 kHz	120	500 mg L^{-1}	100	N/R*	[1]
Methyl orange	Maghemite nanoparticles + H_2O_2 + AMF	H = 16 kA m^{-1}; f = 200 kHz	120	500 mg L^{-1}	98.43	68.23	[13]
Textile industry wastewater	Maghemite nanoparticles + H_2O_2 + AMF	H = 16 kA m^{-1}; f = 200 kHz	120	500 mg COD L^{-1}	47.79	24.09	[13]
Landfill leachate	Maghemite nanoparticles + H_2O_2 + AMF	H = 58 kA m^{-1}; f = 100 kHz	120	6560 mg COD L^{-1}	90.21	74.73	[13]
Polyethylene microplastics	Maghemite nanoparticles + H_2O_2 + AMF	H = 58 kA m^{-1}; f = 100 kHz	120	1000 mg L^{-1}	N/R*	80	[14]
Methylene blue	NanoPET/NF + H_2O_2 + AMF	H = 24 kA/m; f = 200 kHz	350	100 mg L^{-1}	40.04	N/R*	[15]
Methylene blue	MicroPET/SC + H_2O_2 + AMF	H = 24 kA/m; f = 200 kHz	120	100 mg L^{-1}	51.56	N/R*	This work

*N/R = Not reported.

References

1. Gallo-Cordova, A.; Veintemillas-Verdaguer, S.; Tartaj, P.; Mazar, E.; Morales, M.P.; Ovejero, J.G. Engineering Iron Oxide Nanocatalysts by a Microwave-Assisted Polyol Method for the Magnetically Induced Degradation of Organic Pollutants. *Nanomaterials* **2021**, *11*, 1052, doi:https://doi.org/10.3390/nano11041052.

2. Ügdüler, S.; Van Geem, K.M.; Denolf, R.; Roosen, M.; Mys, N.; Ragaert, K.; De Meester, S. Towards Closed-Loop Recycling of Multilayer and Coloured PET Plastic Waste by Alkaline Hydrolysis. *Green Chem.* **2020**, *22*, 5376–5394, doi:https://doi.org/10.1039/d0gc00894j.

3. Tabassum, S.; Debnath, P.; Ahmed, F.; Bashar, M.S.; Akhtar, U.S.; Ahmed, S.; Hossain, S. Sustainable Synthesis of Ni-Based Metal – Organic Framework from Waste Plastic as a Potential Electrochemical Sensor for Detecting Heavy Metals. *ACS Sustain. Resour. Manag.* **2026**, *3*, 1097–1111, doi:https://doi.org/10.1021/acssusresmgt.5c00650.

4. Claudio A. Téllez S., Eduardo Hollauer, M.A Mondragon, V.M.C. Fourier Transform Infrared and Raman Spectra, Vibrational Assignment and Ab Initio Calculations of Terephthalic Acid and Related Compounds. *Spectrochim. Acta - Part A Mol. Biomol. Spectrosc.* **2000**, *2*, 993–1007, doi:https://doi.org/10.1016/S1386-1425(00)00428-5.

5. Pritchina, E.A.; Kolesov, B.A. Raman Spectra of Terephthalic Acid Crystals in the Temperature Range 5 K–300 K. *Spectrochim. Acta - Part A Mol. Biomol. Spectrosc.* **2018**, *202*, 319–323, doi:https://doi.org/10.1016/j.saa.2018.05.065.

6. Lee, M.W.; Kim, M.S.; Kim, K. Infrared and Raman Spectroscopic Study of Terephthalic Acid Adsorbed on Silver Surfaces. *J. Mol. Struct.* **1997**, *415*, 93–100, doi:https://doi.org/10.1016/S0022-2860(97)00081-1.

7. Čolnik, M.; Pečar, D.; Knez, Ž.; Goršek, A.; Škerget, M. Kinetics Study of Hydrothermal Degradation of PET Waste into Useful Products. *Processes* **2022**, *10*, 24, doi:https://doi.org/10.3390/pr10010024.

8. López, J.; Rey, A.; García-Araya, J.F.; Álvarez, P.M. Green Synthesis of Magnetite-Based Catalysts for Solar-Assisted Catalytic Wet Peroxide Oxidation. *Catalysts* **2022**, *12*, 271, doi:https://doi.org/10.3390/catal12030271.

9. Herrera-Aquino, R.; Nuñez, N.; Magro, R.; Veintemillas-Verdaguer, S.; Winkler, E.L.; Espinosa, A.; Morales, M.P.; Gallo-Cordova, A. Designed to Heat and React: Fast Microwave-Engineered Iron Oxide Nanoflowers with Controlled Anisotropy for Magnetic Induction-Assisted Oxidation Processes. *ACS Appl. Nano Mater.* **2026**, *9*, 9497–9512, doi:https://doi.org/10.1021/acsanm.6c01066.

10. Hernández-Rodríguez, E.A.; García-Espinoza, J.D.; Treviño-Resendez, J.; Razo-Negrete, M.; Acosta-Santoyo, G.; Godínez, L.A.; Robles, I. Magnetic Field-Assisted Electro-Fenton System Using Magnetite as a Sustainable Iron Source for Wastewater Treatment. *Processes* **2026**, *14*, 264,

doi:https://doi.org/10.3390/pr14020264.

11. Centro Elia, B.; Morales Ovalle, M.A.; Franco, V.G.; Fuentes García, J.A.; Goya, G.F. Magnetically Enhanced Fenton-like Processes by Nanofibers: Real-Time Observation of Tetracycline Degradation in Pig Manure Wastewater. *Environ. Technol. (United Kingdom)* **2026**, *47*, 1478–1492, doi:https://doi.org/10.1080/09593330.2025.2607666.

12. Wang, D.; Chen, T.; Jiang, Y.; Cai, X.; Li, Y.; Chen, Y.; Yang, G.; Pan, F. Alternating Magnetic Field Accelerates the Transformation between Fe(II) and Fe(III) of Fe@NiF$_{e2}$O$_4$ in a Fenton-Like Process. *J. Environ. Eng.* **2024**, *150*, doi:https://doi.org/10.1061/joeedu.eeeng-759.

13. Gallo-Cordova, A.; Castro, J.J.; Winkler, E.L.; Lima, E.; Zysler, R.D.; Morales, M.P.; Ovejero, J.G.; Streitwieser, D.A. Improving Degradation of Real Wastewaters with Self-Heating Magnetic Nanocatalysts. *J. Clean. Prod.* **2021**, *308*, 127385, doi:https://doi.org/10.1016/j.jclepro.2021.127385.

14. Gallo-Cordova, A.; Corrales-Pérez, B.; Cabrero, P.; Force, C.; Veintemillas-Verdaguer, S.; Ovejero, J.G.; Morales, M.P. Magnetic Harvesting and Degradation of Microplastics Using Iron Oxide Nanoflowers Prepared by a Scaled-up Procedure. *Chem. Eng. J.* **2024**, *490*, 151725, doi:https://doi.org/10.1016/j.cej.2024.151725.

15. Herrera-Aquino, R.; Rivera, F.L.; Díaz-Ufano, C.; Veintemillas-Verdaguer, S.; Morales, M.P.; Gallo-Cordova, A. Magnetic Capture and Process-Oriented Upcycling of PET Nanoplastics Enabled by Mesocrystalline Iron Oxide Nanoflowers. *Chem. Eng. J.* **2026**, *546*, 179081, doi:https://doi.org/10.1016/j.cej.2026.179081.
